# Myeloid Zinc Finger 1 (Mzf1) Differentially Modulates Murine Cardiogenesis by Interacting with an *Nkx2.5* Cardiac Enhancer

**DOI:** 10.1371/journal.pone.0113775

**Published:** 2014-12-01

**Authors:** Stefanie A. Doppler, Astrid Werner, Melanie Barz, Harald Lahm, Marcus-André Deutsch, Martina Dreßen, Matthias Schiemann, Bernhard Voss, Serge Gregoire, Rajarajan Kuppusamy, Sean M. Wu, Rüdiger Lange, Markus Krane

**Affiliations:** 1 Department of Experimental Surgery, Department of Cardiovascular Surgery, Deutsches Herzzentrum München, Technische Universität München (TUM), Munich, Germany; 2 Institute for Medical Microbiology, Immunology and Hygiene, Technische Universität München (TUM), Munich, Germany; 3 Clinical Cooperation Groups “Antigen-specific Immunotherapy” and “Immune-Monitoring”, Helmholtz Center Munich (Neuherberg), TUM, Munich, Germany; 4 Cardiovascular Research Center, Division of Cardiology, Harvard Medical School, Department of Medicine, Massachusetts General Hospital, Boston, Massachusetts, United States of America; 5 Division of Cardiovascular Medicine, Stanford Cardiovascular Institute, Stanford University School of Medicine, Stanford, California, United States of America; 6 Institute for Stem Cell Biology and Regenerative Medicine, Stanford University School of Medicine, Stanford, California, United States of America; 7 DZHK (German Center for Cardiovascular Research) – partner site Munich Heart Alliance, Munich, Germany; New York Medical College, United States of America

## Abstract

Vertebrate heart development is strictly regulated by temporal and spatial expression of growth and transcription factors (TFs). We analyzed nine TFs, selected by *in silico* analysis of an *Nkx2.5* enhancer, for their ability to transactivate the respective enhancer element that drives, specifically, expression of genes in cardiac progenitor cells (CPCs). *Mzf1* showed significant activity in reporter assays and bound directly to the *Nkx2.5* cardiac enhancer (*Nkx2.5* CE) during murine ES cell differentiation. While Mzf1 is established as a hematopoietic TF, its ability to regulate cardiogenesis is completely unknown. *Mzf1* expression was significantly enriched in CPCs from *in vitro* differentiated ES cells and in mouse embryonic hearts. To examine the effect of *Mzf1* overexpression on CPC formation, we generated a double transgenic, inducible, tetO*Mzf1-Nkx2.5* CE eGFP ES line. During *in vitro* differentiation an early and continuous *Mzf1* overexpression inhibited CPC formation and cardiac gene expression. A late *Mzf1* overexpression, coincident with a second physiological peak of *Mzf1* expression, resulted in enhanced cardiogenesis. These findings implicate a novel, temporal-specific role of *Mzf1* in embryonic heart development. Thereby we add another piece of puzzle in understanding the complex mechanisms of vertebrate cardiac development and progenitor cell differentiation. Consequently, this knowledge will be of critical importance to guide efficient cardiac regenerative strategies and to gain further insights into the molecular basis of congenital heart malformations.

## Introduction

The understanding of underlying principles in cardiogenesis is crucial to identify pathophysiological mechanisms involved in congenital heart disease and to gain further insights into the molecular basis for a cardiac regenerative therapy [1]–[3]. Vertebrate heart development is strictly regulated by temporal- and spatial-restricted expression of different growth and transcription factors (TFs) [1], [2]. Several cardiac progenitor cell populations, which have been characterized by the expression of different TFs or defined by the activity of specific enhancer elements using transgenic models, are involved in the developmental processes that guide cardiogenesis [3]–[6]. In our study we focused on a murine cardiac progenitor cell (CPC) population defined by the activity of an *Nkx2.5* cardiac enhancer (*Nkx2.5* CE) element located about 9 kb upstream of the *Nkx2.5* start codon [3], [7]. This CPC population has been described to represent the first identifiable heart-forming cell population in the developing mouse embryo [3].

The myeloid zinc finger protein 1 (Mzf1) is a *Krüppel* class zinc finger TF preferentially expressed in hematopoietic stem cells, myeloid progenitor cells, as well as in differentiated myeloid cells [8]-[10]. Mzf1 is associated with hematopoiesis as transcriptional regulator in committing hematopoietic precursor cells to a myeloid fate, especially for granulopoiesis [8], [11], [12]. Additionally, several reports also suggest a role of Mzf1 in tumorigenesis influencing cell migration and invasion [13]–[16]. Mzf1 has thirteen zinc finger motifs arranged in two different DNA binding domains which recognize the consensus sequences 5′ AGTGGGGA 3′ (zinc fingers 1–4) and 5′ CGGGNGAGGGGGAA 3′ (zinc fingers 5–13) [8], [11]. Mzf1 can act as transcriptional activator or inhibitor in a context dependent manner as shown for a subset of different cell lines [8].

In this study we analyzed nine candidate TFs, selected by *in silico* analysis of the *Nkx2.5* CE, with a known background in embryonic cardiogenesis or hemangiogenesis, for their ability to transactivate the *Nkx2.5* CE element [3], [7]. We found, that *Mzf1* displayed an impressive activation of *Nkx2.5* CE in luciferase reporter assays and we were able to demonstrate specific binding of *Mzf1* to the *Nkx2.5* CE. In support of a potential role of *Mzf1* in cardiac development, we could show that *Mzf1* is highly expressed in embryonic CPCs *in vivo*. To dissect the role of *Mzf1* in cardiac differentiation, we generated a doxycyclin inducible *Mzf1* overexpressing murine *Nkx2.5* CE eGFP ES cell line and examined the differential effects of *Mzf1* on CPC formation. Interestingly, *Mzf1* was able to either repress or enhance cardiogenesis in a temporal-specific manner as indicated by the frequency of eGFP^+^ cells and the degree of cardiac gene expression. Thus, our findings support a novel bi-phasic role of *Mzf1* during embryonic heart development.

## Materials and Methods

Methods are described briefly. Please find a detailed methods section in the online supporting information (Methods S1).

### Luciferase Reporter Assays

Cells (HEK 293, H9c2, HL-1 and NFPE) were seeded in 24-well plates and grown to 70–80% confluence. HEK 293 and H9c2 cells are commercially available at ATCC (Manassas, VA). HL-1 cells were a kind gift of Prof. Dr. William Claycomb [17]. NFPE cells were a kind gift of Prof Dr. Karl-Ludwig Laugwitz but are also commercially available at ATCC. Each well of cells was co-transfected with four plasmids: the expression plasmid (pcDNA3.1(−) containing the candidate cDNA; 150 ng), a pCMV β-Gal plasmid (to normalize transfection efficiency, 50 ng), the pBluescript KSII(+) (250 ng, to normalize the quantity of DNA used in each transfection) and a promoterless pGL3 basic reporter plasmid containing the 2.5 kb fragment of the *Nkx2.5* CE including the base promoter [3] in front of a luciferase gene (150 ng). In further experiments additional mutant forms of the pGL3-*Nkx2.5* CE BP plasmid were used (mutation of the Mzf1 and Mesp1 binding sites). The empty pcDNA3.1 was used as a negative control in all assays. After 48 h cells were lysed, luciferase activity was determined and normalized to β-galactosidase activity. Each transfection experiment was performed in triplicate in at least three independent experiments.

### Electromobility Shift Assays (EMSA)

Proteins (Mzf1, Mesp1) were translated *in vitro* by a TNT T7-coupled reticulocyte lysate system (Promega, Madison, WI). Pairs of complementary Cy5- or Cy3-tagged oligonucleotides were annealed overnight and binding reactions were performed with 5-10 µl of *in vitro* translated protein (Mzf1, Mesp1; confirmed by Western Blot with specific antibodies) or the same amount of unprogrammed reticulocyte lysate (RL) as a negative control. For competition assays unlabeled specific competitor (same sequence as the Cy5-tagged probes, 10- and 50-fold excess) and mutant competitor (10-fold excess) were added to test for specifity of DNA binding. Three independent experiments were performed.

### Chromatin Immunoprecipitation (ChIP) Assays

Cross-linking of day 9 differentiated *Nkx2.5* CE eGFP ES cells (a kind gift of Dr. Sean M Wu [3]) was achieved by incubating the cells with 1% formaldehyde for 30 min at RT. For subsequent sonication cell-lysates were diluted and DNA was sheared. Precleared cell-lysates were then incubated with an antibody against Mzf1 or an isotype-matched control and pulled down by protein A/G-Sepharose beads. Immune complexes were washed extensively with buffer (increasing stringency) and eluted by boiling in SDS sample buffer. DNA purification was performed and with the primer sets #1 (forward 5′ TAC CGG CAG AGA CTG AAG TTT 3′, reverse 5′ ATT AGT GTG AAC ACA ACA CTC G 3′ corresponding to -9340 to -9220 of the *Nkx2.5* CE, fragment size 121 nt), #2 (forward 5′ AAG CTT GGC GTG TGA CAT TGT 3′, reverse 5′ GAT TGT GAA CCG GTA GGC GG 3′ corresponding to -9123 to -8921 of the *Nkx2.5* CE, fragment size 203 nt), #3 (forward 5′ TGA GCG CCG CCG TTT ATG CT 3′, reverse 5′ GAT GGA TCC GAT GGG AGC TG 3′ corresponding to -8360 to -8246 of the *Nkx2.5* CE, fragment size 114 nt) and #4 (forward 5′ AAA TCA ATC ACA GCC CCA AGT G 3′, reverse 5′ GTT TAT GGA AAA CTC AAA TAG CAG 3′, corresponding to −8235 to −8048 of the *Nkx2.5* CE, fragment size 188 nt) the appearance of specific parts of the *Nkx2.5* CE was validated. The precipitation of background DNA was controlled by an amplification with primers against *β-Actin* (fragment size 97 nt, primer sequence see Table S2). PCRs were performed with equal volumes of Mzf1 chip'd samples and the corresponding IgG control on a Thermo Cycler (Bio-Rad, Munich, Germany) and 35 cycles.

### Site Directed Mutagenesis

All mutant forms of the pGL3-*Nkx2.5* CE BP plasmid were constructed by long polymerase chain reaction-based techniques using the QuikChange Multi Site-Directed Mutagenesis Kit with *PfuTurbo* polymerase (Stratagene, La Jolla, CA) and different primers containing the desired mutations (for two putative Mzf1 and two Mesp1 binding sites). After amplification all methylated and hemimethylated DNA was digested with the restriction enzyme *Dpn*I followed by a transformation of the remaining mutated single stranded DNA into XL10 Gold ultracompetent cells. The correct DNA sequence of all constructs was confirmed by DNA sequencing.

### Animals

Mice were housed in an accredited facility in compliance with the European Community Directive related to laboratory animal protection (2010/63/EU). All transgenic mouse lines have been described in detail previously. For extraction of embryos or organs mice were first anesthetized with isoflurane (2-chloro-2-(difluoromethoxy)-1,1,1-trifluoro-ethane) and then euthanized by cervical dislocation. Embryos of the *Nkx2.5* CE eGFP transgenic mice [3] were collected on E 9.5 from timed matings (a positive mating plug indicates E 0.5). Mouse embryos were used for FACS analysis as described in the respective sections. *αMHC*-Cre/ROSA26^mT/mG^ transgenic mice [18] were provided for heart extraction for FACS analysis as described in the respective section. All animal experiments, like organ or embryo extractions, were performed in accordance with the European regulations for animal care and handling (2010/63/EU) and were approved by the Regierung von Oberbayern.

### Lentiviral transduction of ES cells

We used a previously described doxycyclin inducible lentiviral tet-on expression system [19] (a kind gift of Dr. K. Hochedlinger) modified with an IRES puromycin element. The murine complete *Mzf1* cDNA tagged by a flag sequence was subcloned into the modified pLvtetO backbone in front of the IRES element. Lentivirus production by 293 cells was previously described by Gregoire et al. [20]. For transduction the virus containing supernatant was collected after 48 h, filtered and then directly used without further concentration.

A doxycyclin inducible tetO*Mzf1*-*Nkx2.5* CE eGFP ES cell line was established by co-transducing *Nkx2.5* CE eGFP ES cells with tetO*Mzf1*-IRES-puromycin and rtTA lentiviral particles (approved by the Regierung von Oberbayern, Az. 50-8791-26.384.1776). Antibiotic selection was performed with doxycyclin (dox) and puromycin. About 10 days post-transduction, colonies were individually expanded and scored by qRT-PCR for sufficient expression of *Mzf1*. Morphology and growing behavior of the transduced cell lines were virtually indistinguishable from untreated murine ES cells.

### Flow cytometry

Single cell suspension was prepared from *Nkx2.5* CE eGFP and dox-inducible tetO*Mzf1-Nkx2.5* CE eGFP differentiating ES cells. *Nkx2.5* CE eGFP mouse embryos were dissected on E 9.5 and single cell suspension was obtained. Adult cardiomyocytes (CMs) were isolated from six week old *αMHC*-Cre/ROSA26^mT/mG^ mice. Dead cells were stained with propidium iodide solution for flow cytometry.

### RNA Isolation and qRT-PCR

Total RNA was isolated and reverse transcribed into first strand cDNA. Semiquantitative real time PCR (qRT-PCR) was performed using gene-specific primer sets (Table S2) for 40 cycles. ΔCT calculations were performed and each sample was normalized against its β-actin value.

### Data Analysis and Statistical Analysis

All assays were at least performed in triplicates. Data are presented as mean values ± standard error (S.E.M.). Statistical differences were evaluated using the unpaired Student's t-test or the Mann-Whitney-U test. Comparison of several groups was done by one way ANOVA or the Kruskal-Wallis test on ranks including appropriate post-hoc tests. A value of *p* <0.05 was considered to be statistically significant. In all figures statistical significance is indicated as follows: * *p* <0.05 and ** *p* <0.01.

## Results

An *in silico* transcription factor (TF) binding site analysis [21] (P-Match [22] http://www.gene-regulation.com/cgi-bin/pub/programs/pmatch/bin/p-match.cgi, PROMO 3.0 http://alggen.lsi.upc.es/recerca/menu_recerca.html, JASPAR database http://jaspar.genereg.net, ConSite http://phylofoot.org/consite, TFSEARCH ver. 1.3 http://www.cbrc.jp/research/db/TFSEARCH.html) of the cardiac specific *Nkx2.5* enhancer [7] (−9641 to −7540 bp upstream of the murine *Nkx2.5* transcriptional initiation site) and the base promoter [3] (−520 to −24 bp upstream of the ATG of the *Nkx2.5* gene) revealed a couple of candidates for potential interaction (Table S1). Nine of these TFs (*Gata4* [7], *Hand1* [23], *Sox17* [24], *Klf4* [25], *Elk1* [26], *Msx1* [27], *Mzf1*, *Brachyury* [28], *Mesp1* [29]) which are known in the context of embryonic heart development or hemangiogenesis were selected for further analysis.

### Mzf1 strongly activates the *Nkx2.5* CE using different cell lines

**Figure 1:**
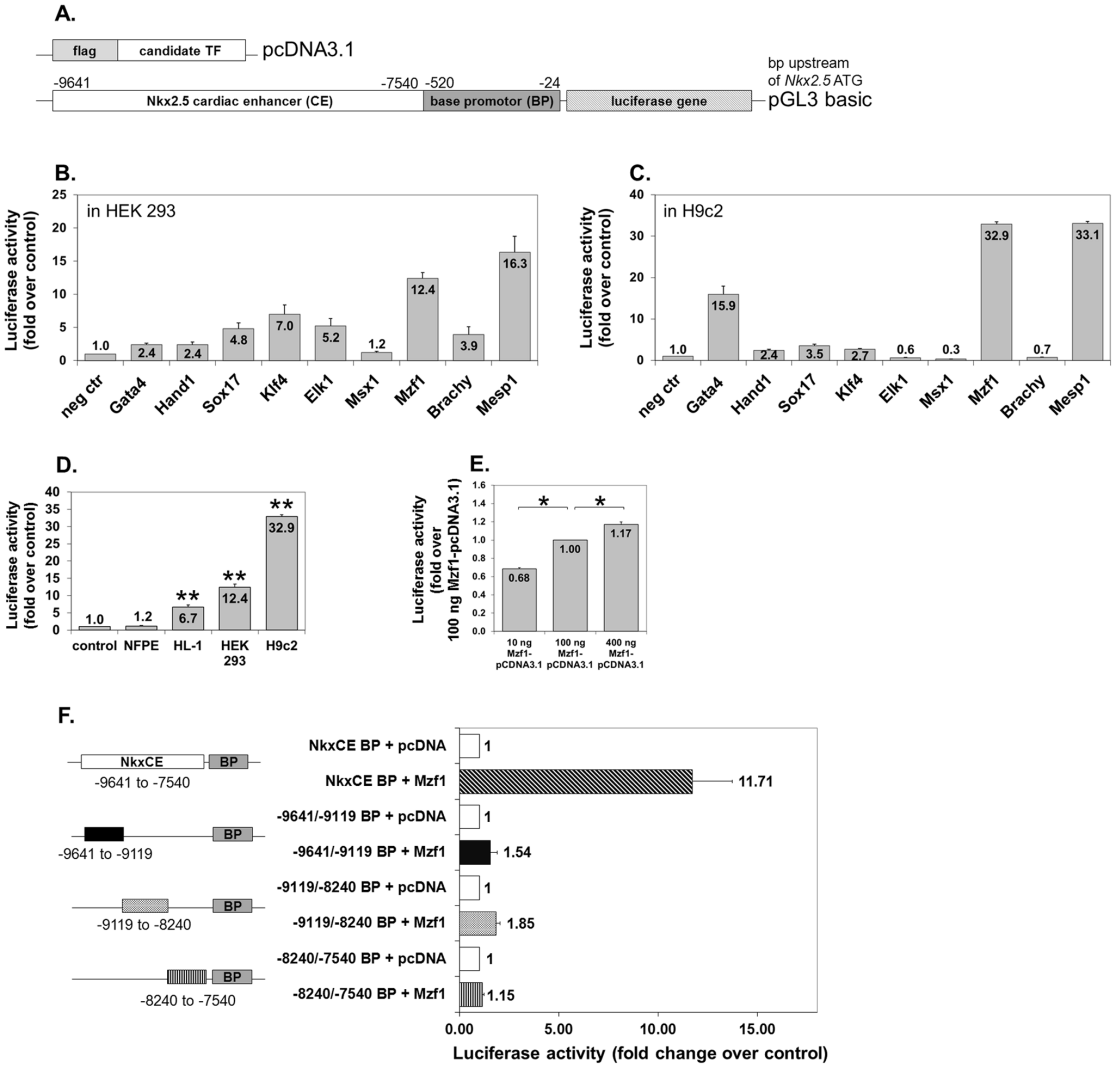
TF screening on the *Nkx2.5* CE element by luciferase reporter assays. **A.** Plasmid constructs for luciferase reporter assays. The empty modified pcDNA3.1 was used as a negative control in all assays. **B.** TF screening by luciferase assays using HEK 293 cells (human embryonic kidney fibroblasts). Fold change is compared to the negative control (neg ctr) (pcDNA3.1). **C.** TF screening by luciferase assays using H9c2 cells (rat myoblasts). Fold change is compared to the negative control (neg ctr) (pcDNA3.1). **D.** *Mzf1* activates the *Nkx2.5* CE element in atrial HL-1 cells but not in endothelial NFPE cells. Asterisks indicate a significant difference compared to the control (pcDNA3.1); **  =  p <0.01. **E.** Dose dependent effect of *Mzf1*-pcDNA3.1-DNA on *Nkx2.5* CE activation in HEK 293 cells; *  =  p <0.05. **F.** Effect of truncating different parts of the *Nkx2.5* CE according to Lien and co-workers [7] on luciferase activation by *Mzf1* in HEK 293 cells.

Luciferase reporter assays were performed to evaluate the activation capacity of the candidate TFs on the *Nkx2.5* CE. Each TF was inserted into a modified pcDNA3.1 vector and co-transfected with the *Nkx2.5* CE luciferase reporter plasmid (Fig. 1A) in HEK 293 cells. A more than 5-fold activation of the luciferase gene was found for *Elk1*, *Klf4*, *Mesp1* and *Mzf1* (Fig. 1B).

To determine if this effect is cell line specific, luciferase assays were also performed in H9c2, a rat myoblastic cell line corroborating a strong (more than 30-fold) transgene activation by *Mesp1* and *Mzf1* (Fig. 1C). Further luciferase assays using murine atrial HL-1 cells confirmed the induction of luciferase expression by *Mzf1* (Fig. 1D) and *Mesp1* (Fig. S1A). Interestingly no transactivation of either factor occurred in endothelial NFPE cells (Fig. 1D, Fig. S1A). Luciferase induction occurred in a dose-dependent fashion for both TFs in HEK 293 cells (Fig. 1E, Fig. S1B).

Our results confirmed the findings reported by Bondue et al. [29] regarding the interaction between *Mesp1* and the cardiac specific *Nkx2.5* CE element. However, we were intrigued by the as yet unknown function of *Mzf1* during cardiomyogenesis. Hence, we chose to further explore the role of *Mzf1* in this context.

### Mzf1 shows activating effects on different parts of the *Nkx2.5* CE

According to Lien et al. [7] the identified *Nkx2.5* CE consists of activating and non-activating elements regarding the potential to drive a cardiac specific β-galactosidase expression. Corresponding to their description we investigated the potential of *Mzf1* and *Mesp1* to induce luciferase expression driven by different parts of the *Nkx2.5* CE (−9641 to −9119; −9119 to 8240; −8240 to −7540). Luciferase activity was significantly reduced following *Mzf1* and *Mesp1* treatment when the *Nkx2.5* CE was truncated, regardless which part of the *Nkx2.5* CE was used for the assay, suggesting the presence of activating binding sites in each portion of the *Nkx2.5* CE (Fig. 1F, Fig. S1C). Furthermore, it could be possible that one of the truncated fragments contains a binding site of Mzf1, while the others contain binding sites for essential co-factors.

### Mzf1 directly binds to the *Nkx2.5* CE

Since *in silico* analysis of the *Nkx2.5* CE revealed between 19 and 92 potential binding sites for Mzf1 (Table S1) distributed all over the *Nkx2.5* CE, we decided to focus on two binding motifs similar to the well-known zinc finger motifs already described by other researchers [8], [11].

**Figure 2:**
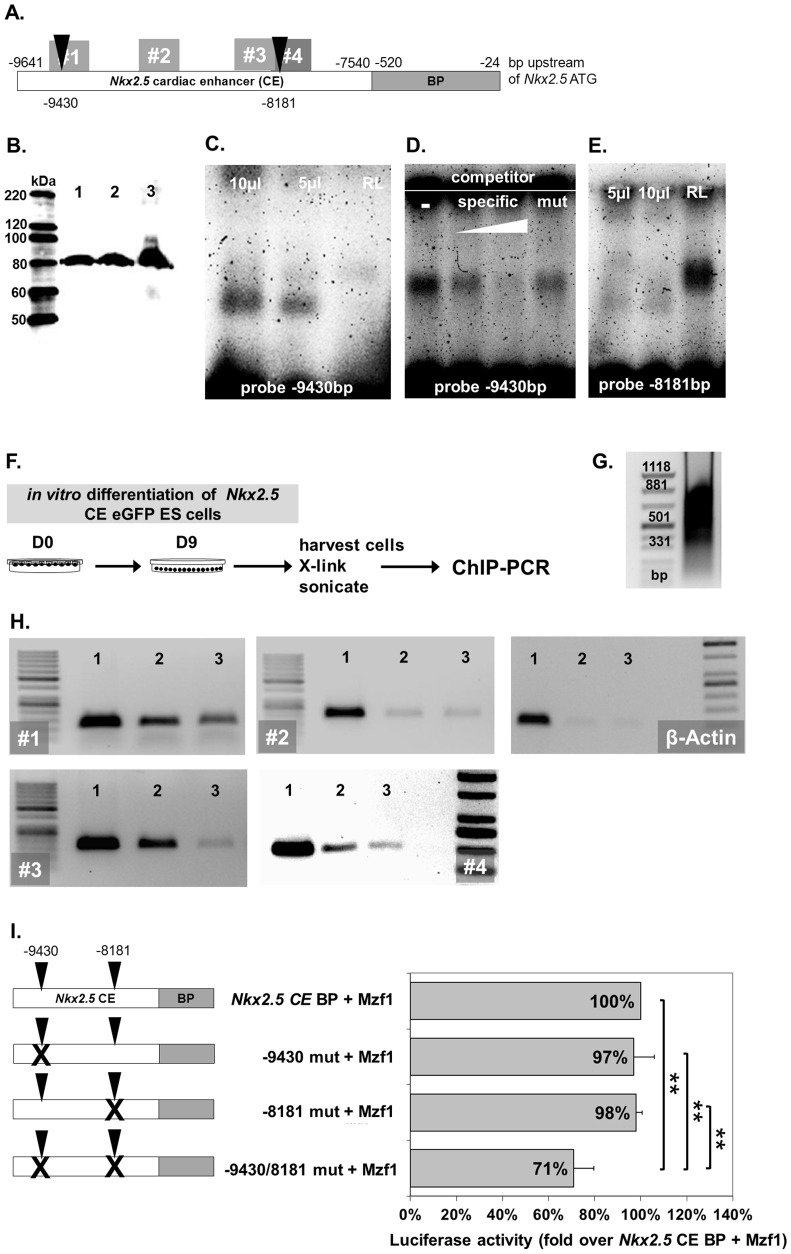
Direct binding of Mzf1 to the *Nkx2.5* cardiac enhancer *in vitro* and *in vivo*. **A.** Locations of analyzed described Mzf1 binding motifs in the *Nkx2.5* CE [8], [11] (black triangles) and primer sets #1-#4 for ChIP PCR (grey rectangles with numbers). **B.** *In vitro* translated Mzf1 protein from the flag-*Mzf1*-pcDNA3.1 confirmed by an anti-flag antibody in western-blotting (lane 3). As a control whole cell lysates from 293 cells transfected with the flag-*Mzf1*-pCDNA3.1 plasmid were used (lane 1 & 2). The predicted molecular weight for Mzf1 is 84 kDa. **C.** Different amounts of *in vitro* translated Mzf1 (10µl, 5µl) bound to the *Nkx2.5* CE at the binding motif corresponding to zinc fingers 5-13 (black triangle at position -9430 bp) [8], [11] in an electromobility shift assay (EMSA). Unprogrammed reticulocyte lysate (RL) was applied as a control. **D.** Competition assays with untagged mutant (mut, 10-fold excess) and specific probes (10- and 50-fold excess) were performed to ensure specificity. **E.** *In vitro* binding to the motif corresponding to zinc fingers 1-4 (at position −8181 bp) [8], [11] by EMSA could not be confirmed. Different amounts of *in vitro* translated Mzf1 (10µl, 5µl) were used. Unprogrammed reticulocyte lysate (RL) was applied as a control. **F.** Experimental set-up for ChIP assays. Chromatin was isolated from day nine differentiated *Nkx2.5* CE eGFP ES cells. **G.** Chromatin was sheared by sonication to obtain fragment sizes between 250 and 1000 bp. **H.** ChIP-PCR on purified chromatin using a polyclonal anti-Mzf1 and an isotype-matched control antibody. Lane 1: 4% sonicated input chromatin. Lane 2: Chromatin precipitated with the Mzf1 antibody. Lane 3: Chromatin precipitated with an IgG matched control antibody. **I.** Effect of mutating two Mzf1-binding sites at positions −9430 bp and -8181 bp in the *Nkx2.5* CE on luciferase activity by *Mzf1* in HEK 293 cells (**  =  p <0.01).

The binding of Mzf1 to the *Nkx2.5* CE could be confirmed by electromobility shift assays (EMSA) using the core Mzf1 binding motif 5′-AGGGGGA-3′ (corresponding to the zinc fingers 5–13, [8], [11]) at position −9430 bp of the *Nkx2.5* CE using Cy5-tagged probes and *in vitro* translated Mzf1 protein (Fig. 2A–C). Competition assays with untagged mutant (10-fold excess) and specific probes (10- and 50-fold excess) were performed to ensure specificity of the binding reaction at position −9430 (Fig. 2D). However, binding to the motif 5′-GTGGGGA-3′ (corresponding to the zinc fingers 1–4, [8], [11]) at position −8181 bp of the *Nkx2.5* CE could not be approved by EMSA (Fig. 2E). Direct binding of *in vitro* translated Mesp1 to the *Nkx2.5* CE was also confirmed by EMSA (Fig. S1D–F).

To further corroborate Mzf1 binding to the *Nkx2.5* CE *in vivo* ChIP assays with a polyclonal anti-Mzf1 antibody were performed on cross-linked murine day nine differentiated *Nkx2.5* CE eGFP ES cells followed by PCR analysis (Fig. 2F). Chromatin shearing led to fragment sizes between 250 and 1000 bp (Fig. 2G). Binding of Mzf1 on day nine of *in vitro* differentiation of murine ES cells could be validated with primer set #3 corresponding to a 114 nt fragment at position −8360 to −8246 of the *Nkx2.5* CE (Fig. 2A+H) and primer set #4 corresponding to a 188 nt fragment at position −8235 to −8048 of the *Nkx2.5* CE (Fig. 2A+H). Primer set #1 (−9340 to −9220) led to a darker band in the sample precipitated with the anti-Mzf1 antibody compared to the control but the background was very strong for this primer set (Fig. 2H). No binding could be confirmed with primer set #2 (−9123 to −8921) (Fig. 2H). A background control with primers against *β-Actin* approved that precipitation of unspecific DNA was low (Fig. 2H).

In a next step site directed mutagenesis was performed on the pGL3-*Nkx2.5* CE BP plasmid to mutate the analyzed binding sites of Mzf1 (Fig. 2I) and also Mesp1 (Fig. S1G). Subsequent luciferase assays with the *Mzf1*-pcDNA3.1 could not show a reduced luciferase activity when only one, either at position −9430 bp or −8181 bp, of the binding sites in the *Nkx2.5* CE was mutated (Fig. 2I). However, a combined mutation of both binding sites led to a significant reduction of about 30% of luciferase activity (Fig. 2I). For *Mesp1* we could confirm the significance of the binding site at position −29 bp by a significant reduction of luciferase activity by 26% (Fig. S1G) when this site was mutated as already indicated by ChiP-assays by Bondue and co-workers [29]. No reduction of luciferase activity could be shown for a mutation of the Mesp1 binding site at position −9138 bp, despite ChiP-assays demonstrated binding of Mesp1 to this site [29].

### Mzf1 shows biphasic kinetics during *in vitro* differentiation of murine ES cell lines

**Figure 3:**
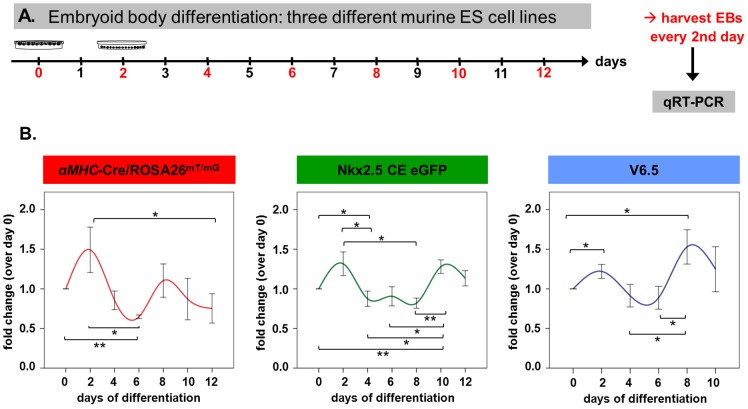
Biphasic kinetics of *Mzf1* expression during *in vitro* differentiation. **A.** Experimental set-up for *in vitro* differentiation assays of three different murine ES cell lines for the evaluation of time-dependent *Mzf1* expression levels. **B.** *Mzf1* expression levels showed a biphasic course during *in vitro* differentiation of *αMHC*-Cre/ROSA26^mT/mG^ -, *Nkx2.5* CE eGFP - and V6.5 ES cells; *  =  p <0.05; **  =  p <0.01.

Next we analyzed the kinetics of *Mzf1* mRNA expression during ES cell *in vitro* differentiation. Three different murine ES cell lines (V6.5 ES, the transgenic *Nkx2.5* CE eGFP ES [3] and the transgenic *αMHC*-Cre/ROSA26^mT/mG^ ES [18]) were studied every other day starting from day 0 of differentiation (when hanging drops are prepared) for the expression of *Mzf1* (for experimental set-up see Fig. 3A). We found a clear biphasic mRNA expression pattern of *Mzf1* in all of the three ES cell lines with an early peak around day two and a second peak between day eight and day ten of *in vitro* differentiation (Fig. 3B).

### Mzf1 gene expression is upregulated in CPCs but not in adult cardiomyocytes

As previously described, luciferase reporter assays indicated an activation of the *Nkx2.5* CE element by *Mzf1*. Additionally, specific binding of Mzf1 to the *Nkx2.5* CE element could be confirmed by EMSA and ChIP assays.

**Figure 4:**
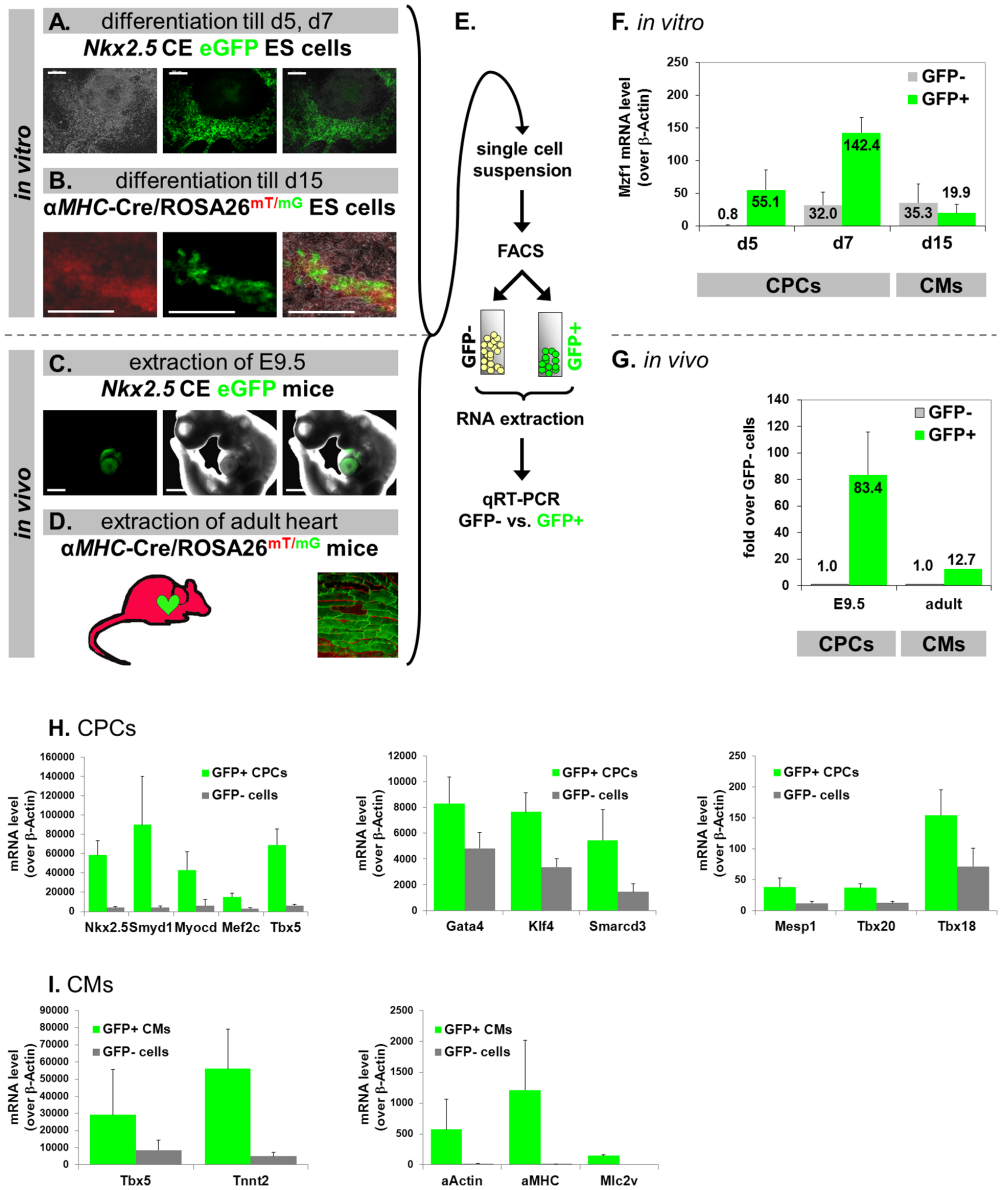
Differential expression of *Mzf1* in cardiac progenitor cells (CPCs) but not in cardiomyocytes (CMs) and gene expression profiles of *in vitro* differentiated CPCs and CMs. Scale bars: 200 µm for all panels, except C: 500 µm. **A.-D.** Detection of CPCs and mature CMs by activation of eGFP expression. Illustration of transgenic cell lines (**A.–B.**) and animal models (**C.–D.**). **E.** Experimental set-up for isolating eGFP-positive and -negative cell populations by FACS. **F.** Gene expression analysis after FACS sorting of *in vitro* differentiated *Nkx2.5* CE eGFP ES cells (**A.**) revealed a considerable up-regulation of *Mzf1* in eGFP^+^ CPCs but not for mature CMs (**B.**) compared to the respective eGFP^−^ cells. **G.** Correspondingly, *Mzf1* expression was up-regulated in eGFP^+^ CPCs isolated from E 9.5 embryos (**C.**) but not to a comparable amount in mature CMs isolated from postnatal (> 3 weeks) hearts (**D.**) compared to the respective eGFP^−^ cells. **H.** *Nkx2.5* CE eGFP ES cells were differentiated till day 5–7. GFP positive (CPCs) and GFP negative cells were sorted by FACS. Gene expression profiles of typical cardiac developmental marker genes (*Nkx2.5, Mef2c, Gata4, Tbx20*, etc.) were evaluated by qRT-PCR. **I.** *αMHC*-Cre/ROSA26^mT/mG^ ES cells were differentiated till day 15. GFP positive (CMs) and GFP negative cells were sorted by FACS. Gene expression profiles of typical cardiac and sarcomeric marker genes (*Tnnt2*, *αMHC*, etc.) were evaluated by qRT-PCR.

We postulated that if Mzf1 interacts with the *Nkx2.5* CE *in vivo* it should also be differentially expressed within *Nkx2.5* CE eGFP positive CPCs (Fig. 4A). To examine this hypothesis, we differentiated *Nkx2.5* CE eGFP ES cells for either five or seven days. During *in vitro* differentiation of this cell line first eGFP positive CPCs usually emerge on day five. EGFP-positive CPCs and eGFP-negative cells were then isolated by fluorescence activated cell sorting (FACS) on day five and seven. Both cell populations were lysed for total RNA extraction and subsequent gene expression analysis by qRT-PCR (Fig. 4E). We observed that CPCs expressed a considerably higher level of *Mzf1* than non-CPCs, on day five and seven of *in vitro* differentiation (Fig. 4F). These isolated eGFP positive CPCs further exhibit high expression levels of typical early cardiac marker genes compared to eGFP negative non-CPCs (Fig. 4H).

To further confirm the role of *Mzf1* in *Nkx2.5* CE positive CPCs *in vivo*, time-pregnant transgenic *Nkx2.5* CE eGFP mice were dissected on day E 9.5 where eGFP expression and thus *Nkx2.5* CE activity peaks during embryonic development [3]. Whole embryos were digested by a collagenase mixture to obtain single cell suspension for accomplishing FACS. As analyzed by qRT-PCR *Mzf1* expression in eGFP positive cells, which exclusively correspond to the E 9.5 heart (Fig. 4C) was more than 80-fold upregulated when compared to the level in embryonic eGFP negative cells (Fig. 4G).

To determine the relative expression of *Mzf1* in more mature cardiomyocytes, we utilized the *αMHC*-Cre/ROSA26^mT/mG^ [18] transgenic murine ES cell line for further experiments. Mature cardiomyocytes (CMs) expressing *αMHC* switch from red to green fluorescence which is induced by Cre-mediated excision of the td-tomato expression cassette (Fig. 4B). GFP positive CMs were isolated by FACS on day 15 of differentiation. In contrast to CPCs the more mature eGFP positive CMs do not show an elevated level of *Mzf1* compared to the eGFP negative population (Fig. 4F). A more detailed gene expression profile of isolated CMs including typical cardiac and sarcomeric markers is presented in Fig. 4I.

Furthermore, also isolated eGFP positive CMs from postnatal hearts of the *αMHC*-Cre/ROSA26^mT/mG^ transgenic mice (> three weeks of age), do not show a similar elevation over non-cardiomyocytes when compared to E 9.5 CPCs (Fig. 4G).

### Mzf1 gain-of-function studies modify CPC number during ES differentiation

**Figure 5:**
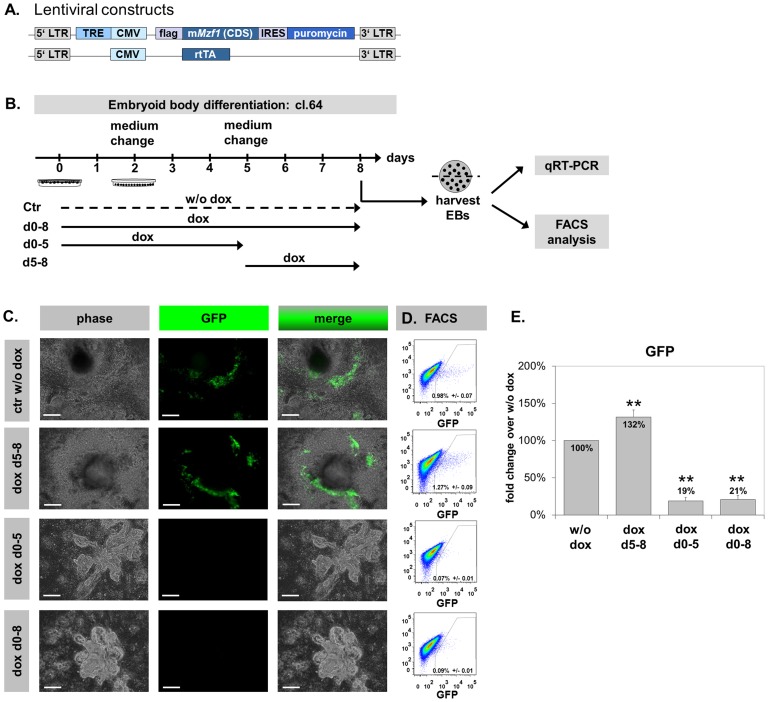
*In vitro* differentiation of the double-transgenic dox-inducible *Mzf1* overexpressing tetO*Mzf1*-*Nkx2.5* CE eGFP ES cell line. Scale bars: 200 µm for all panels. **A.** Lentiviral constructs for the production of the doxycyclin-inducible tetO*Mzf1*-*Nkx2.5* CE eGFP ES line. LTR: long terminal repeats. TRE: tetracyclin responding element. CMV: cytomegalovirus promoter. IRES: internal ribosomal entry site. rtTA: reverse tetracyclin transactivator. **B.** *In vitro* differentiation protocols with time-schedules of dox-treatment. **C.** Morphology of differentiating EBs on day eight. Permanent and day 0–5 dox-treatment led to closely packed globular clusters. In contrast dox-treatment from day 5 showed a normal differentiation pattern comparable to the control w/o dox. **D/E.** FACS analysis revealed a significant increase in eGFP^+^ CPCs for dox-treatment from day 5 of differentiation whereas a continuous and day 0-5 dox-treatment resulted in a significant decrease of eGFP^+^ CPCs compared to control w/o dox; **  =  p <0.01.

Next, to directly address the effect of *Mzf1* on CPCs and on cardiac differentiation in general, we generated a double-transgenic, doxycyclin (dox) inducible *Mzf1* over-expressing murine ES cell line by lentiviral transduction of the transgenic *Nkx2.5* CE eGFP ES cell line (tetO*Mzf1*-*Nkx2.5* CE eGFP ES). A plasmid driving dox-inducible expression of *Mzf1* and puromycin resistance separated by an internal ribosome entry site (IRES) was co-transduced with a plasmid that constitutively expresses a reverse tetracycline transactivator (rtTA) (Fig. 5A, Fig. S2A). Sufficient inducibility of *Mzf1* expression was confirmed in three cell lines (clones 42, 44 and 64; Fig. S2B). Furthermore, it was proofed that *Mzf1* overexpression decreased steadily after stopping dox supplementation of the medium (Fig. S2C–D). Two days after dox-removal the *Mzf1*-mRNA-level was more than 50% reduced compared to the starting level. And after four days the *Mzf1*-mRNA-level was not different from the samples without dox-addition. Morphology (Fig. S2E), pluripotency (Fig. S2F, anti Sox2 immunostaining) and *Mzf1*-expression (p  =  0.242) were comparable between the tetO*Mzf1*-*Nkx2.5* CE eGFP ES cell line without dox treatment, and the parent *Nkx2.5* CE eGFP ES cell line.

*In vitro* differentiation assays of the tetO*Mzf1*-*Nkx2.5* CE eGFP ES cell line were performed by the standard hanging drop method [30] to assess the effects of *Mzf1* overexpression on CPC number by flow cytometry. ES cells were differentiated for eight days. In line with the physiological, biphasic course of *Mzf1*-mRNA expression during ES differentiation (Fig. 3B) doxycyclin was added according to different treatment schedules (Fig. 5B). Besides a permanent *Mzf1*-overexpression by dox-treatment (day 0 - 8), time intervals from zero to five and from five to eight days were analyzed. The tetO*Mzf1-Nkx2.5* CE eGFP ES cell line differentiated without dox treatment (ctr w/o dox) was used as reference.

The appearance of eGFP positive, beating cells at day five to six of *in vitro* differentiation was indistinguishable between the control (w/o dox) and the parent murine *Nkx2.5* CE eGFP ES cells (Fig. S2G, Video S1, S2).

The comparable amount of dead cells between the different approaches (Fig. S2H) identifiable by propidium iodide staining using FACS analysis indicated that the overexpression of *Mzf1* did not influence cell viability during *in vitro* differentiation.

Cell proliferation was additionally controlled by MTT assays. Whereas tetO*Mzf1*-*Nkx2.5* CE eGFP ES cells which grew with dox for 48h were indistinguishable from their untreated counterparts (p  =  0.927), ES cells treated with dox for a longer period (five to nine days) proofed significantly more proliferative (p  =  0.003) (Fig. S2I).

Furthermore, an efficient *Mzf1* overexpression during *in vitro* differentiation assays was confirmed by qRT-PCR (Fig. S3A) and immunostaining with an anti-flag antibody detecting only exogenous Mzf1 (Fig. S3B). The *Mzf1* expression level on day 8 was lower in approaches with permanent dox-treatment than in approaches with late dox-treatment from day 5 during *in vitro* differentiation (Fig. S3A). This may be due to some self-inhibiting mechanisms within *Mzf1* regulation on the mRNA level or due to inactivation of the integrated CMV promoter during *in vitro* differentiation or a combination of both.

*Mzf1* overexpression from day 5 of *in vitro* differentiation showed no morphological differences and also a regular appearance of beating areas compared to the control w/o dox (Fig. 5C, Video S3). In contrast, permanent overexpression of *Mzf1* (day 0–8) and dox treatment from day 0–5 led to severe morphological changes. EBs grew in closely packed, globular clusters while no beating areas could be observed (Fig. 5C).

FACS analysis on day eight of *in vitro* differentiation revealed about 0.98% ± 0.070 eGFP positive cells (CPCs) in the negative control (w/o dox). Interestingly, an overexpression of *Mzf1* from day 5 showed 1.27% ± 0.090 (p  =  0.003) eGFP positive cells depicting an increase of nearly 30% compared to the control (w/o dox) and suggesting an enhancement of cardiogenesis. In contrast, permanent *Mzf1* overexpression significantly reduced the amount of CPCs to 0.085% ± 0.012 eGFP positive cells (p <0.001) indicating a strong inhibitory effect on cardiogenic differentiation. No significant difference of eGFP positive CPCs could be detected between both protocols with an early overexpression of *Mzf1* (day 0–5 and day 0–8) (p  =  0.596) (Fig. 5D+E).

### Mzf1 modifies cardiac gene expression

Cardiac gene expression was analyzed by qRT-PCR to confirm results obtained from flow cytometry in terms of down- or up-regulation of cardiogenesis, respectively.

**Figure 6:**
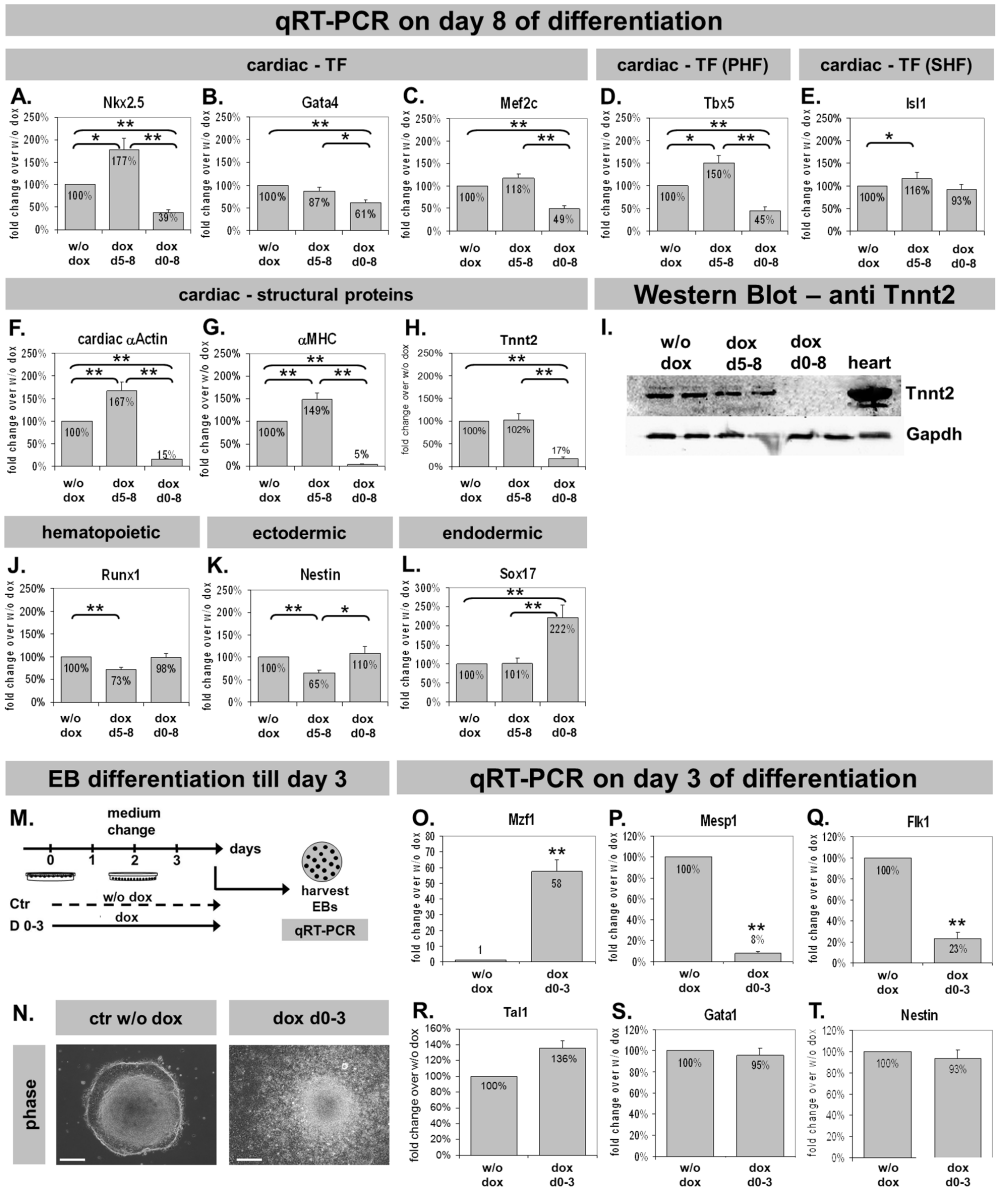
Gene expression analysis during *in vitro* differentiation of tetO*Mzf1*-*Nkx2.5* CE eGFP ES cells. *  =  p<0.05; **  =  p <0.01 for all panels. **A–H.** Expression of selected cardiac genes. PHF: primary heart field. SHF: secondary heart field. **I.** Protein expression by western-blotting for Tnnt2 and Gapdh. **J–L.** Expression of selected hematopoietic (**J.**), ectodermal (**K.**) and endodermal (**L.**) genes. **M.** Experimental set-up for day three *in vitro* differentiation assays. **N.** Morphology of differentiating EBs on day three. Dox-treatment for three days increased cell proliferation. Scale bars indicate 200 µm. **O–T.** Gene expression analysis of *Mzf1* (**O.**), *Mesp1* (mesodermal) (**P.**), *Flk1* (cardiovascular progenitor marker) (**Q.**), *Tal1*, *Gata1* (hematopoietic marker) (**R., S.**) and *Nestin* (ectodermal) (**T.**).

Temporary *Mzf1* overexpression from day five led to a significantly up-regulated *Nkx2.5* expression compared to the control w/o dox (p  =  0.028). In contrast, *Nkx2.5* was dramatically down-regulated by a permanent *Mzf1* overexpression (p <0.001) (Fig. 6A) confirming regulatory effects of *Mzf1* on CPCs. The regulatory effect was also seen for the cardiac TFs *Tbx5*, *Isl1* and *Mef2c* but not for *Gata4* (Fig. 6B–E). Furthermore, cardiac structural genes were significantly repressed by permanent overexpression of *Mzf1* whereas a temporary overexpression led to a significant elevation of cardiac structural genes like *α-MHC* and the pancardiac *α-Actin* (Fig. 6F-G), but not *Troponin T* (*Tnnt2*) (Fig. 6H) (see also western blot, Fig. 6I). The hematopoietic marker *Runx1* [31] was down-regulated by dox-stimulated *Mzf1* expression from day five (p <0.001) but was not affected by a permanent *Mzf1* overexpression (p  =  0.371) (Fig. 6J). Ectodermal and endodermal differentiation was assessed by the expression of *Nestin* and *Sox17* [2], [24], respectively. *Nestin* was down-regulated by *Mzf1*-overexpression from day 5 (p <0.001) but was unaffected by a continuous overexpression (p  =  0.779) (Fig. 6K). Interestingly, *Sox17* was unaffected by a late *Mzf1* overexpression (p  =  0.975) but a permanent *Mzf1* overexpression led to a significant increase over the control w/o dox (p <0.001) (Fig. 6L).

To directly address a cardiac specific inhibition by early *Mzf1* overexpression we arranged a different experimental set-up for further *in vitro* differentiation assays (Fig. 6M). The dox-inducible tetO*Mzf1-Nkx2.5* CE eGFP ES cell line was differentiated for only three days with or without addition of dox. Figure 6N shows that EBs grew faster under permanent dox-treatment for three days which is in agreement with the increased cell proliferation of tetO*Mzf1*-*Nkx2.5* CE eGFP ES cells that grew with dox for more than 48 h (Fig. S2I). On day three EBs were harvested and total RNA was applied to qRT-PCR. First, *Mzf1* expression was confirmed by qRT-PCR showing a 58-fold overexpression by dox-treatment compared to untreated control (p <0.001, Fig. 6O). Next, we analyzed marker genes involved in early cardiac development, such as *Mesp1*, an early cardiac mesoderm marker [32] or *Flk1* known as an early marker of cardiovascular commitment [33]. *Mesp1* as well as *Flk1* were considerably down-regulated by *Mzf1* overexpression (p <0.001, Fig. 6P-Q), confirming the already assumed inhibition of cardiogenesis by an early over-expression of *Mzf1*. Interestingly, *Tal1* (also known as *Scl*), typically expressed in hemangioblasts (progenitor cells of the hematoendothelial lineage, [34]), as well as *Gata1*, a marker of the hematopoietic lineage [35], and *Nestin* (ectodermal marker), were not affected by an early *Mzf1* over-expression for three days (Fig. 6R-T).

## Discussion

The specification and differentiation of pluripotent stem cells *in vitro* and *in vivo* is driven by a complex transcriptional regulatory network. Most of the evidence about the TF *Mzf1* and its impact on other genes are exclusively based on *in vitro* luciferase assays and EMSA [8], [36]. Herein we studied, comprehensively, the role of *Mzf1* on the frequency of cardiac progenitor cells using an *Nkx2.5* cardiac specific enhancer element. We identified for the first time that *Mzf1* can activate the *Nkx2.5* CE in several cell lines and that Mzf1 binds directly to the *Nkx2.5* CE both *in vitro* and *in vivo*.

Our diverging results of the *Nkx2.5* CE activation by *Mzf1* in different cell lines indicates that *Mzf1* can act in a cell specific manner as previously implied by Morris and co-workers [8] for hematopoietic (K562, Jurkat) or nonhematopoietic cell lines (NIH 3T3, 293). Interestingly, *Mzf1* is able to transactivate the *Nkx2.5* CE in muscular and cardiac cell lines such as H9c2 and HL-1 but not in endothelial cell lines such as NFPE cells. This suggests that the mechanism of *Mzf1* transcription is dependent on the presence of tissue-specific regulators or differential protein modifications that affect Mzf1 function as postulated previously [8]. Most likely, tissue-specific co-factors are necessary for an appropriate function within a cellular system, (e.g. YY1 acts together with Gata4 in CPCs [20]). Our finding that Mzf1 interacts with the *Nkx2.5* CE raises the possibility that the binding of Mzf1 to the *Nkx2.5* CE may require the presence of other *Nkx2.5* CE-bound TFs [8], [11].

We also found a biphasic pattern of *Mzf1* expression during *in vitro* differentiation of murine ES cell lines potentially indicating a dual mode of action during lineage specification. Other factors like Myf-6 [37] or D-mef2 [38] that influence lineage specification also act in a biphasic manner during embryonic development.

Our hypothesis that Mzf1 plays a role in cardiogenesis via an interaction with the *Nkx2.5* CE was further supported by the differential expression of *Mzf1* in purified *Nkx2.5* CE positive CPCs at days five and seven of differentiation as well as in mouse embryonic hearts at E 9.5 but to a much lower extent in mature adult cardiomyocytes. These results indicate that the main influence of Mzf1 on *Nkx2.5* CE labelled CPCs takes place during early cardiomyocyte differentiation but not after terminal differentiation of these cells.

Since *Mzf1* appears to regulate gene expression in CPCs, we examined the effect of *Mzf1* overexpression using a murine tetO*Mzf1*-*Nkx2.5* CE eGFP ES cell line. Flow cytometry results clearly indicated an increased frequency of CPCs induced by an *Mzf1* overexpression from day five of *in vitro* differentiation. In contrast, continuous overexpression of *Mzf1* from day 0-8 resulted in significant reduction of CPC formation. We furthermore found evident morphological changes during differentiation under permanent dox-addition. Settled EBs showed globular clusters which were closely packed while no beating areas could be observed. It can be assumed that the permanent *Mzf1* overexpression led to a different migration behavior of cells in these EBs since it is well known that *Mzf1* plays a role in migration and invasion [13]–[16]. However, *Mzf1* overexpression from day 5 exhibited an EB-morphology typical for undirected murine ES-cell differentiations and a regular appearance of beating areas. Based on this observation, we concluded that *Mzf1* overexpression can induce cardiac lineage expansion in a temporal-specific fashion.

**Figure 7:**
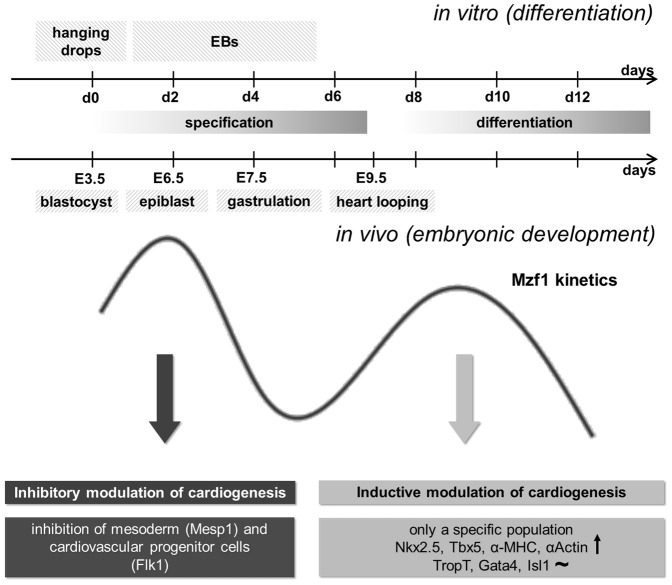
Potential mechanistic role of *Mzf1* during embryonic development. The first peak of physiological *Mzf1* up-regulation occurs during specification of pluripotent cells, corresponding to the epiblast stage during murine development on E 6.0 or 6.5. At this time *Mzf1* seems to have an inhibitory effect on cardiac lineage commitment. The second physiological peak of *Mzf1* expression occurs during differentiation of pluripotent cells around day eight of *in vitro* differentiation. An overexpression of *Mzf1* at the beginning of this peak resulted in stimulation of cardiogenesis.

Taken together, our results implicate a role for *Mzf1* in the control of cardiac commitment by an interaction with the *Nkx2.5* cardiac enhancer. As *Mzf1* was significantly enhanced in a CPC population *in vitro* as well as in embryonic heart tissue and late overexpression of *Mzf1* promoted cardiac lineage commitment we propose that *Mzf1* may be a novel regulator of embryonic heart development. Figure 7 summarizes the physiological biphasic kinetics of *Mzf1* expression. The first peak of *Mzf1* up-regulation occurs early during specification of pluripotent cells: Around day two of *in vitro* differentiation, corresponding with the epiblast stage during murine development on E 6.0 or 6.5. At this time *Mzf1* seems to have an inhibitory effect on cardiac lineage commitment as shown by our results (down-regulation of *Mesp1*). *Mzf1* may inhibit the generation of cardiac mesoderm by suppressing *Mesp1* and *Flk1* expression. *Runx1* (hematopoietic) and *Nestin* (ectodermal) are virtually unaffected by a permanent overexpression of *Mzf1*. The second physiological peak of *Mzf1* expression occurs during differentiation of pluripotent cells around day eight of *in vitro* differentiation. An overexpression of *Mzf1* at the beginning of this peak (from day 5), in parallel with the endogenous upregulation of the *Nkx2.5* expression which is initiated at day four of *in vitro* differentiation and is highly increased at day five to seven [3], results in a moderate stimulation of cardiogenic commitment. Besides *Nkx2.5*, typical cardiac primary heart field (PHF) genes like *Tbx5*, sarcomeric genes like *αMHC* or the pancardiac structural marker *cardiac αActin* are significantly up-regulated.

*Mzf1* transcriptional regulation mechanisms seem to be tissue-specific as well as stage dependent. The divergent findings of stimulation or repression of specific marker genes by time-dependent *Mzf1* overexpression supported earlier suggestions that *Mzf1* might be necessary for a normal differentiation program involving a balance between positive and negative regulatory signals [36].

A global deletion of *Mzf1* in the mouse did not lead to embryonic lethality nor did the authors mention evident alterations during heart development [10]. It could be speculated that a loss of Mzf1 during development may be compensated by another transcription factor as it is known for Mesp1 and Mesp2 during the early stages of gastrulation [39]. However, we have to assume that the role of *Mzf1* in heart development is more stabilizing or modulating than actually stimulating.

In summary, the findings that *Mzf1* can simultaneously activate or repress specific genes following time-dependent *Mzf1* overexpression support a modulatory role for *Mzf1* in normal cardiac development where a proper balance between positive and negative regulatory signals is critical. Further investigation of the role of *Mzf1* in cardiac development *in vivo* may provide novel insights into molecular mechanisms of vertebrate heart development, which are crucial for devising successful cardiac regenerative therapies in the future.

## Supporting Information

Figure S1 Luciferase reporter assays and EMSA for Mesp1 on the *Nkx2.5* cardiac enhancer element. **S1A.** Besides in HEK 293 and H9c2 cells *Mesp1* activated the *Nkx2.5* CE element in atrial HL-1 cells but not in endothelial NFPE cells. Asterisks indicate significance compared to the control (pcDNA3.1); **  =  p <0.01. **S1B.** Dose reduction of *Mesp1*-pcDNA3.1-DNA significantly decreased luciferase activity in 293 cells; *  =  p <0.05. **S1C.** Skipping parts of the *Nkx2.5* CE according to Lien and co-workers [7] led to significant reduction of luciferase activation by *Mesp1* in HEK 293 cells. **S1D.** Locations of confirmed Mesp1 binding sites on the *Nkx2.5* CE [29] (black triangles). **S1E.** *In vitro* translated Mesp1 protein from the flag-*Mesp1*-pcDNA3.1 confirmed by an anti-flag antibody in western-blotting (lane 2). As a control whole cell lysate from 293 cells transfected with the flag-*Mesp1*-pCDNA3.1 plasmid was used (lane 1). The predicted molecular weight for Mesp1 is 37 kDa. **S1F.** *In vitro* translated Mesp1 (10 µl) bound to an E-Box-motif (black triangle at position -29 bp in the *Nkx2.5* CE) [29] in an electromobility shift assay (EMSA). The same amount of unprogrammed reticulocyte lysate (RL) was applied as a control. Competition assays with untagged mutant (mut, 10-fold excess) and specific (10-fold excess) probes were performed to ensure specificity. *In vitro* binding to another E-Box-motif in the *Nkx2.5* CE (at position -9138 bp) [29] could not be confirmed. **S1G.** Effect of mutating two Mesp1-binding sites at positions -9138 bp and -29 bp in the *Nkx2.5* CE BP on luciferase activity by *Mesp1* in HEK 293 cells (**  =  p <0.01).
(TIF)

Figure S2 Generation and verification of a double-transgenic dox-inducible *Mzf1* overexpressing *Nkx2.5* CE eGFP ES cell line and characterization of the tetO*Mzf1*-*Nkx2.5* CE eGFP ES cell line compared to the parent *Nkx2.5* CE eGFP ES cells. S2A. Transduction of *Nkx2.5* CE eGFP ES cells with *Mzf1* and rtTA lentiviruses. Scale bars: 200 µm. **S2B.** Dox-inducible expression of *Mzf1* could be confirmed in three expanded clones (cl. 42, 44 and 64). **S2C.+D.** Confirmation of sensitivity for dox-inducible *Mzf1* expression in clone 64. **S2E.** The morphology of tetO*Mzf1*-*Nkx2.5* CE eGFP ES cells with and w/o dox was undistinguishable from the parent *Nkx2.5* CE eGFP ES cells. Scale bars: 200 µm for all panels. **S2F.** Pluripotency of tetO*Mzf1*-*Nkx2.5* CE eGFP ES cells with and w/o dox was evaluated by immunostaining with an anti-Sox2 antibody. As a control the parent *Nkx2.5* CE eGFP ES cells were also stained. The negative controls were performed with the secondary antibody only. Scale bars: 200 µm for all panels. **S2G.** The morphology of differentiated tetO*Mzf1*-*Nkx2.5* CE eGFP ES cells w/o dox was comparable to the parent differentiated *Nkx2.5* CE eGFP ES cells on day seven of *in vitro* differentiation. Scale bars: 200 µm for all panels. **S2H.** A negative effect of permanent or temporary dox-treatment and by this *Mzf1* overexpression on differentiating tetO*Mzf1*-*Nkx2.5* CE eGFP ES cells was excluded by comparison of the amount of dead cells (evaluated by propidiumiodide staining during flow cytometry) between the different approaches. **S2I.** Cell proliferation was evaluated by MTT assays in tetO*Mzf1*-*Nkx2.5* CE eGFP ES cells treated with dox for different time intervals (control was w/o dox); **  =  p <0.01.
(TIF)

Figure S3 *Mzf1* upregulation in the tetO*Mzf1*-*Nkx2.5* CE eGFP ES cell differentiation assays with different dox-treatment schedules. **S3A.** Verification of *Mzf1* upregulation on day eight of *in vitro* differentiation of tetO*Mzf1*-*Nkx2.5* CE eGFP ES cells by qRT-PCR; *  =  p <0.05; **  =  p <0.01. **S3B.** Co-staining with an anti-flag and an anti-GFP antibody (AB) to detect exogenous overexpression of Mzf1 (red fluorescence) in day 7 differentiated tetO*Mzf1*-*Nkx2.5* CE eGFP ES cells and cardiac progenitor cells (green fluorescence). Scale bars: 200 µm for all panels.
(TIF)

Methods S1 Detailed methods section.
(DOCX)

Table S1 Transcription factor candidates from *in silico* analysis of the *Nx2.5* CE element in alphabetical order. Boldly printed TFs were chosen for further analysis by luciferase reporter assays.
(DOCX)

Table S2 Sequences of primer-sets for gene expression analysis by qRT-PCR.
(DOCX)

Video S1 *In vitro* differentiation of the parent *Nkx2.5* CE eGFP ES cell line. Time-lapse imaging of *Nkx2.5* CE eGFP EBs on day eight of *in vitro* differentiation. The video is not real-time but assembled from single pictures photographed in a time series. Therefore beating frequency is an artifact of exposure time. The video displays 50–70 images. Magnification is 100×.
(MPG)

Video S2 *In vitro* differentiation of tetO*Mzf1*-*Nkx2.5* CE eGFP ES cells w/o dox led to eGFP positive beating areas undistinguishable from the parent *Nkx2.5* CE eGFP ES cell line. Time-lapse imaging of tetO*Mzf1*-*Nkx2.5* CE eGFP EBs on day eight of *in vitro* differentiation w/o dox. The video is not real-time but assembled from single pictures photographed in a time series. Therefore beating frequency is an artifact of exposure time. The video displays 50-70 images. Magnification is 100×.
(MPG)

Video S3 *In vitro* differentiation of tetO*Mzf1*-*Nkx2.5* CE eGFP ES cells with dox from day 5 led to eGFP positive beating areas undistinguishable from the parent *Nkx2.5* CE eGFP ES cell line. Time-lapse imaging of tetO*Mzf1*-*Nkx2.5* CE eGFP EBs on day eight of *in vitro* differentiation with dox from day 5. The video is not real-time but assembled from single pictures photographed in a time series. Therefore beating frequency is an artifact of exposure time. The video displays 50–70 images. Magnification is 100×.
(MPG)
