# Supplementary figures and images for: Myeloid Zinc Finger 1 (Mzf1) Differentially Modulates Murine Cardiogenesis by Interacting with an Nkx2.5 Cardiac Enhancer
Source: PLoS One. 2014 Dec 1;9(12):e113775. doi: 10.1371/journal.pone.0113775 (PMC4249966; doi:10.1371/journal.pone.0113775)

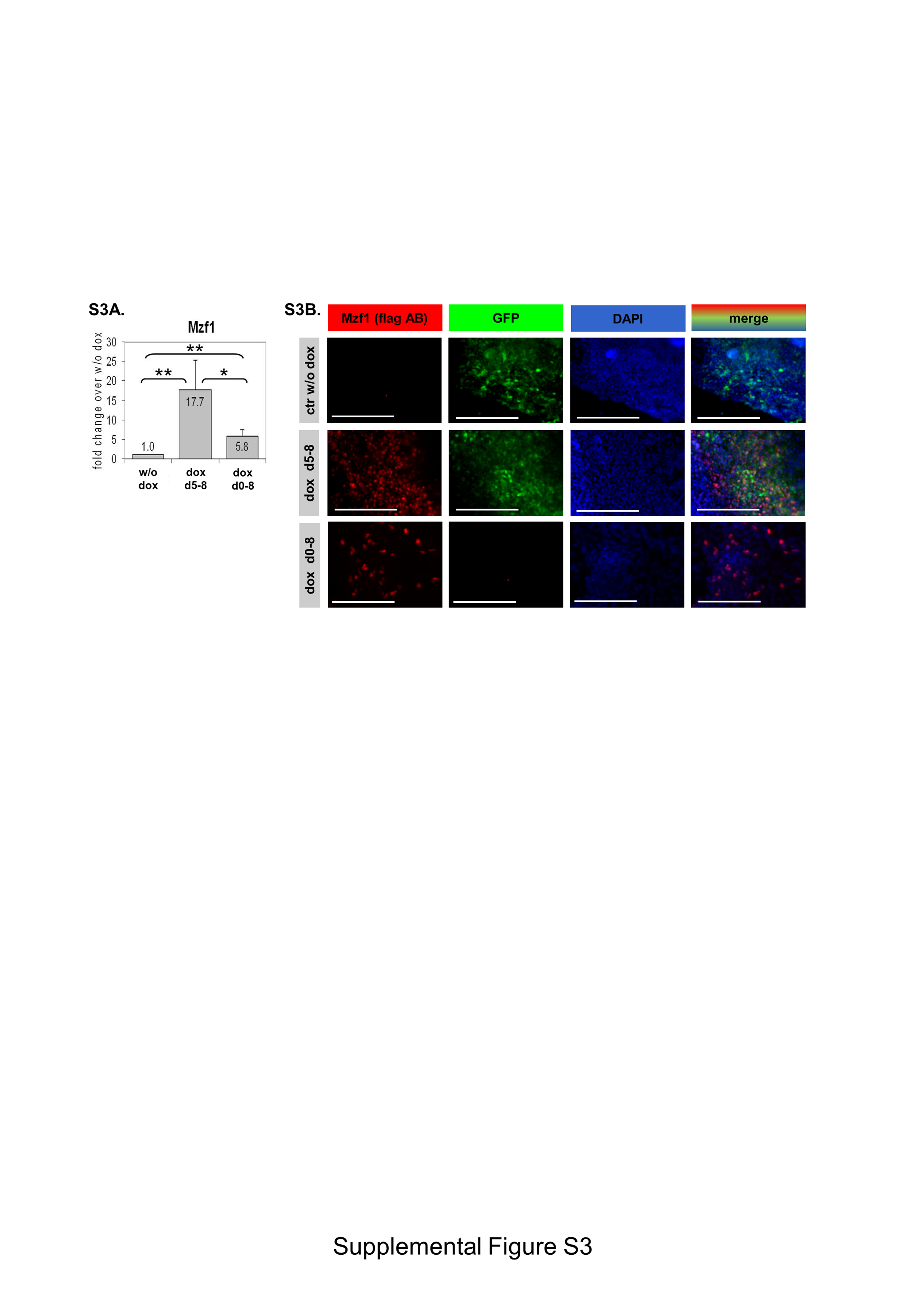

Supplement: Figure S3 — Mzf1 upregulation in the tetOMzf1-Nkx2.5 CE eGFP ES cell differentiation assays with different dox-treatment schedules. S3A. Verification of Mzf1 upregulation on day eight of in vitro differentiation of tetOMzf1-Nkx2.5 CE eGFP ES cells by qRT-PCR; * = p <0.05; ** = p <0.01. S3B. Co-staining with an anti-flag and an anti-GFP antibody (AB) to detect exogenous overexpression of Mzf1 (red fluorescence) in day 7 differentiated tetOMzf1-Nkx2.5 CE eGFP ES cells and cardiac progenitor cells (green fluorescence). Scale bars: 200 µm for all panels. (TIF) [file pone.0113775.s003.tif]
