# Supplementary material for: Myeloid Zinc Finger 1 (Mzf1) Differentially Modulates Murine Cardiogenesis by Interacting with an Nkx2.5 Cardiac Enhancer
Source: PLoS One. 2014 Dec 1;9(12):e113775. doi: 10.1371/journal.pone.0113775 (PMC4249966; doi:10.1371/journal.pone.0113775)
Supplement: Methods S1 — Detailed methods section. (DOCX) [file pone.0113775.s004.docx]

**METHODS S1**

***Cloning of expression plasmids for Luciferase Reporter Assays***

Murine candidate complete cDNAs (*Gata4*, *Hand1, Sox17, Klf4, Elk1, Msx1, Mzf1, Brachyury, Mesp1*) were amplified by PCR with the Fast Start High Fidelity PCR-System, dNTPack (Roche, Mannheim, Germany) from commercially available vectors (imaGenes, now Source BioScience, Nottingham, UK) and subcloned into the pcDNA3.1 (-) (Invitrogen, now Life Technologies, Grand Island, NY) into an *Eco*RI restriction site. The pcDNA3.1 was modified by a flag tag in 5’ direction of the *Eco*RI cloning site.

***Cell culture of 293, H9c2, NFPE and HL-1 cells***

HEK 293 were cultivated in Dulbecco’s Modified Eagle Medium (DMEM) with high glucose (PAA, Pasching, Austria), 5 % FCS (PAA), 100 U/ml : 100 µg/ml penicillin/streptomycin (PAA). The same medium was used for NFPE cells, a kind gift of Prof. Dr. Karl-Ludwig Laugwitz. H9c2 were obtained from ATCC (Manassas, VA) and maintained in DMEM (with high Glucose), 10 % FCS, 100 U/ml : 100 µg/ml penicillin/streptomycin according to vendor’s recommendations. HL-1 cells [[1](#_ENREF_1)] were a kind gift of Prof. Dr. William Claycomb. They were cultured according to his recommendations with supplemented Claycomb medium (Sigma-Aldrich, St. Louis, MO), 10 % FCS (Sigma-Aldrich, #F2442, Batch 058K8426), 100 U/ml : 100 µg/ml penicillin/streptomycin, 2 mM L-Glutamine, 0.1 mM norepinephrine (Sigma-Aldrich) on gelatin/fibronectin coated plates [[2](#_ENREF_2)]. All cells were cultured at 37°C and 5 % CO_2_.

***Luciferase Reporter Assays***

Cells (HEK 293, H9c2, HL-1 and NFPE) were seeded in 24-well plates and grown to 70-80 % confluence. Each well of cells was co-transfected with four plasmids: the expression plasmid (pcDNA3.1(-) containing the candidate cDNA; 150 ng), a pCMV β-Gal plasmid (to normalize transfection efficiency, 50 ng), the pBluescript KSII(+) (250 ng, to normalize the quantity of DNA used in each transfection) and a promoterless pGL3 basic reporter plasmid containing the 2.5 kb fragment of the *Nkx2.5* CE including the base promoter [[3](#_ENREF_3)] in front (5’ direction) of a luciferase gene (150 ng) using FUGENE® HD Transfection Reagent according to the manufacturer’s recommendations (Promega, Madison, WI). To introduce the four plasmids into H9c2 cells SuperFect transfection reagent® (Qiagen, Hilden, Germany) was used. The empty pcDNA3.1 was used as a negative control in all assays. After 48 h cells were lysed with Reporter Lysis Buffer (Promega), luciferase activity of the cleared supernatant was determined using a Luminometer Lumat LB 9507 (Berthold Technologies, Bad Wildbad, Germany) and normalized to β-galactosidase activity (measured with CPRG (Roche) as the substrate and chemiluminescence was detected using an ELISA reader MR 5000 at a wavelength of 595nm (Dynex Technologies, Sullyfield Circle Chantilly, VA)). Each transfection experiment was performed in triplicate in at least three independent experiments.

***Electromobility Shift Assays (EMSA)***

Proteins (Mzf1, Mesp1) were translated *in vitro* by a TNT T7-coupled reticulocyte lysate system (Promega, Madison, WI) using the pcDNA3.1-flag-*Mzf1* or pcDNA3.1-flag-*Mesp1* expression vector as DNA template. Pairs of complementary Cy5- or Cy3-tagged oligonucleotides (*Mzf1_-9430*: forward 5’ Cy5-GAC AAA AGA TGT CCC CCT ACC TAA AGA TA 3’; reverse 5’ Cy5-GTA TCT TTA GGT AGG GGG ACA TCT TTT GT 3’; *Mzf1_-8181:* forward 5’ Cy5-TTT TGG AAA TAT CCC CAC CAC CAA AAG CA; reverse 5’ Cy5-GTG CTT TTG GTG GTG GGG ATA TTT CCA AA; *Mesp1_-29*: forward 5’ Cy5-CTC TCT GCT ACC CAC CTG GCC GGA TCC GA 3’, reverse 5’ Cy5-TT CGG ATC CGG CCA GGT GGG TAG CAG AGA 3’; *Mesp1_-9138*: forward 5’ Cy3-CTG CAG CCG TCA TGT GCA CCT TGA AAG CT 3’, reverse 5’ Cy3-AAG CTT TCA AGG TGC ACA TGA CGG CTG CA 3’; binding sites underlined, TIB Molbiol, Berlin, Germany) were annealed in a buffer containing 10 mM Tris-HCl (pH 8.0), 50 mM NaCl, and 1 mM EDTA overnight. The binding reactions were performed in binding buffer (10 mM Tris-HCl pH 7.5, 50 mM KCl, 50 mM NaCl, 1 mM MgCl_2_, 1 mM EDTA, 5 % Glycerol with 5 mM DTT (Promega)) in a total volume of 20 µl containing also 10 ng Poly dI-dC (Sigma Aldrich) and 5-10 µl of *in vitro* translated protein or the same amount of unprogrammed reticulocyte lysate (RL) as a negative control. The reactions were incubated for 15 min at 20°C. For competition assays unlabeled specific competitor (same sequences as the Cy5- or Cy3-tagged probes, in 10- and 50-fold excess, corresponding to 4 and 20 pM of unlabeled competitor DNA) and mutant competitor (*Mzf1_-9430*: forward mutated 5’-GAC AAA AGA TGT AAA AAT ACC TAA AGA TA-3’, reverse mutated 5’-GTA TCT TTA GGT ATT TTT ACA TCT TTT GT-3’, *Mesp1_-29*: forward mutated 5’-CTC TCT GCT ACC TGA AGT GCC GGA TCC GA-3’, reverse mutated 5’-TT CGG ATC CGG CAC TTC AGG TAG CAG AGA-3’; mutated sites underlined, ELLA Biotech, Munich, Germany) in 10-fold excess (corresponding to 4 pM of unlabeled competitor DNA) were added to test for specifity of DNA binding. The DNA-protein complexes were separated for 3-4 h by agarose gel (0.35 %) electrophoresis in 0.3 x TAE. Fluorescence was detected at 633 nm (Cy5) or 532 nm (Cy3) by a Typhoon Scanner (GE Healthcare, Chalfont St Giles, Buckinghamshire, UK). Pictures were analyzed with Image Quant TL v2005 (GE Healthcare). Three independent experiments were performed.

***Murine ES cell lines and ES cell culture***

The *Nkx2.5* cardiac enhancer eGFP ES cell line (*Nkx2.5* CE eGFP ES) is a reporter cell line for CPCs [[3](#_ENREF_3)]. Cells begin to express eGFP when the *Nkx2.5* CE is activated, usually between day five and six during *in vitro* differentiation. The *αMHC*-Cre/ROSA26^mT/mG^ ES cell line is a reporter cell line for terminally differentiated cardiomyocytes (CMs) [[4](#_ENREF_4)]. At baseline, the undifferentiated *αMHC*-Cre/ROSA26^mT/mG^ ES cells express membrane-tethered tandem dimerized Tomato red (dTomato). Upon differentiation into CMs, the *αMHC*-Cre is expressed resulting in the excision of the dTomato sequence in the ROSA26 locus and the expression of eGFP specifically in cardiomyocytes. During *in vitro* differentiation cells become eGFP positive between day ten and twelve when the *αMHC* promoter is activated. V6.5 ES cells are commercially available (Novus Biologicals, Littleton, CO). As they originate from a 129 x C57BL/6 mating, they have 50 % contribution from each background.

All murine ES cell lines were grown on a monolayer of mitomycin-inactivated mouse embryonic fibroblasts (MEFs) with murine ES cell medium (DMEM with high glucose, 15 % FCS, 200 U/ml : 200 µg/ml penicillin/streptomycin, 2 mM L-glutamine (PAA), 0.1 mM non-essential amino acid solution (Life Technologies), 0.1 mM β-mercaptoethanol (Sigma-Aldrich), and 10^3^ U/ml leukemia inhibitory factor (LIF) (Millipore, Billerica, MA, USA). Cells were refreshed with new medium daily and passaged every two or three days at a 1:4 ratio. For all cell culture experiments with the doxycyclin inducible *Mzf1* overexpressing *Nkx2.5* CE eGFP ES cell line tetracycline free FCS (PAA) was used. ES cells were examined by an Axiovert 200M microscope (Carl Zeiss AG, Jena, Germany) and pictures were taken with an AxioCam MRm (Zeiss) and the AxioVison Rel 4.8 software version 2.0 (Zeiss).

***Differentiation Assays of murine ES cell lines***

All *in vitro* differentiation assays of murine ES cell lines were performed according to Huang & Wu [[5](#_ENREF_5)]. In brief, murine ES cells were prepared for differentiation by growing them on feeder-free gelatin-coated dishes with IMDM (Iscove's Modified Dulbecco's Media, PAA) as a medium base (including 15 % FCS, 200 U/ml : 200 µg/ml penicillin/streptomycin, 2 mM L-glutamine, 0.1 mM monothioglycerol (Sigma Aldrich), and 10^3^ U/ml LIF) for two days. On the day of differentiation hanging droplets (11 µl per droplet) were prepared with a cell concentration of 2*10^5^/ml (about 250 drops per 15 cm plate). Plates are stored upside down till day two when differentiation medium (IMDM, 15 % FCS, 200 U/ml : 200 µg/ml penicillin/streptomycin, 2 mM L-glutamine, 50 µg/ml ascorbic acid (Sigma-Aldrich), 0.1 mM monothioglycerol) was added to the plates after EBs have formed. Pictures were taken with an AxioCam MRm on an Axiovert 200M microscope supported by the AxioVison Rel 4.8 software. Schematic descriptions of *in vitro* differentiation assays and further procedures are provided in the respective figures. Each *in vitro* differentiation assay was performed in triplicate in at least three independent experiments to get reliable results.

***Chromatin Immunoprecipitation (ChIP) Assays***

*Nkx2.5* CE eGFP ES cells were differentiated according to standard protocols. Cross-linking was achieved by incubating the cells with 1 % formaldehyde (Otto Fischar GmbH & Co. KG, Saarbrücken, Germany) for 30 min at RT on day nine of differentiation and quenching the process with 0.125 M glycine at RT. After cells were washed and harvested by centrifugation they were lysed in buffer containing 10 mM Tris-HCl [pH 8.0] (Carl Roth GmbH, Karlsruhe, Germany), 10 mM NaCl (Carl Roth), 3 mM MgCl_2_ (Sigma-Aldrich), 0.5 % IGEPAL (Sigma-Aldrich), and freshly added protease inhibitors (Sigma-Aldrich). For subsequent sonication cell-lysates were diluted in a buffer consisting of 10 mM Tris-HCl [pH 7.5], 10 mM NaCl, 3 mM MgCl_2_, 1 mM CaCl_2_ (Sigma-Aldrich), 4 % IGEPAL, and freshly added protease inhibitors. DNA was sheared by several rounds of sonication (Sonopuls HD2070 equipped with a MS 73 needle, Bandelin, Berlin, Germany). To ascertain equal amounts of chromatin in each sample, cell extracts were split after sonication into identical aliquots. Sonicated cell lysates were pre-adsorbed with protein A/G-Sepharose beads (GE Healthcare) at 4°C for 1 h, and the precleared lysates were then incubated with an antibody against Mzf1 (MZF-1 H-45 X, sc-66991X, Santa Cruz Biotechnology, Santa Cruz, CA [[6](#_ENREF_6)]) or against Ox-LDL (Ox-LDL R-1 H-140, sc-20753, Santa Cruz Biotechnology) as an isotype-matched control at 4°C overnight. Subsequently 200 µl of protein A/G-Sepharose beads were added and incubated for an additional 1 h at 4°C. Immune complexes were washed extensively with buffer (increasing stringency) and eluted by boiling in SDS sample buffer. DNA purification was performed by the DNeasy Blood & Tissue Kit (Qiagen) according to manufacturer’s recommendation. With the primer sets #1 (forward 5’ TAC CGG CAG AGA CTG AAG TTT 3’, reverse 5’ ATT AGT GTG AAC ACA ACA CTC G 3’ corresponding to -9340 to -9220 of the *Nkx2.5* CE, fragment size 121 nt), #2 (forward 5’ AAG CTT GGC GTG TGA CAT TGT 3’, reverse 5’ GAT TGT GAA CCG GTA GGC GG 3’ corresponding to -9123 to -8921 of the *Nkx2.5* CE, fragment size 203 nt), #3 (forward 5’ TGA GCG CCG CCG TTT ATG CT 3’, reverse 5’ GAT GGA TCC GAT GGG AGC TG 3’ corresponding to -8360 to -8246 of the *Nkx2.5* CE, fragment size 114 nt) and #4 (forward 5’ AAA TCA ATC ACA GCC CCA AGT G 3’, reverse 5’ GTT TAT GGA AAA CTC AAA TAG CAG 3’, corresponding to -8235 to -8048 of the *Nkx2.5* CE, fragment size 188 nt) the appearance of specific parts of the *Nkx2.5* CE was validated. The precipitation of background DNA was controlled by an amplification with primers against *β-Actin* (fragment size 97 nt, primer sequence see Supplemental Table S2). PCRs were performed with equal volumes of Mzf1 chip’d samples and the corresponding IgG control on a Thermo Cycler (Bio-Rad, Munich, Germany) and 35 cycles. DNA products were visualized by ethidium bromide staining after electrophoresis on a 2.0 % agarose gel.

***Site Directed Mutagenesis***

All mutant forms of the pGL3-*Nkx2.5* CE BP plasmid were constructed by long polymerase chain reaction-based techniques using the QuikChange Multi Site-Directed Mutagenesis Kit with PfuTurbo polymerase (Stratagene, La Jolla, CA) and different primers containing the desired mutations (for two Mzf1 and two Mesp1 binding sites: Mzf1_-9430mut 5’ CCG AGA GAC AAA AGA TGT **A**C**A** C**A**T ACC TAA AGA TAC AAG GCC ACA 3’; Mzf1_-8181mut 5’ AAT TTT GCG TTT TGG AAA TAT **A**C**A** CA**A** CAC CAA AAG CAC GAG GAA AGT 3’; Mesp1_-9138mut 5’ GAG TGT CTG CAG CCG T**A**A **C**G**C** GCA CCT TGA AAG CTT GG 3’; Mesp1_-29mut 5’ CA CCA CTC TCT GCT ACC **T**A**T** C**C**G GCC GGA TCC GAA TTA G 3’; binding sites underlined, mutated bases in bold, Ella Biotech, Planegg, Germany). After amplification all methylated and hemimethylated DNA was digested with the restriction enzyme *Dpn*I followed by a transformation of the remaining mutated single stranded DNA into XL10 Gold ultracompetent cells. Single *E.coli* clones were picked, grown in LB medium overnight, and plasmid DNA was purified by the Qiaprep Spin Miniprep Kit (Qiagen) according to the manufacturer’s recommendations. The correct DNA sequence of all constructs was confirmed by DNA sequencing.

***Animals***

Mice were housed in an accredited facility in compliance with the European Community Directive related to laboratory animal protection (2010/63/EU). All transgenic mouse lines have previously been described in detail. For extraction of embryos or organs mice were first anesthetized with isoflurane (2-chloro-2-(difluoromethoxy)-1,1,1-trifluoro-ethane) and then euthanized by cervical dislocation. Embryos of the *Nkx2.5* CE eGFP transgenic mice [[3](#_ENREF_3)] were collected on E 9.5 from timed matings (a positive mating plug indicates E 0.5). Mouse embryos were used for FACS analysis as described in the respective sections. *αMHC*-Cre/ROSA26^mT/mG^ transgenic mice [[4](#_ENREF_4)] were provided for heart extraction for FACS analysis as described in the respective section. All animal experiments, like organ or embryo extractions, were performed in accordance with the European regulations for animal care and handling (2010/63/EU) and were approved by the Regierung von Oberbayern.

***Lentiviral transduction of ES cells***

We used a previously described doxycyclin inducible lentiviral tet-on expression system [[7](#_ENREF_7)] (a kind gift of Dr. K. Hochedlinger) modified with an IRES puromycin element. The murine complete *Mzf1* cDNA tagged by a flag sequence at the 5’ end of *Mzf1* was subcloned into the modified pLvtetO backbone in front of the IRES element. Lentivirus production by 293 cells was previously described by Gregoire et al. [[8](#_ENREF_8)]. For transduction the virus containing supernatant was collected after 48 h, filtered (0.45µm) and was then directly used without further concentration.

A doxycyclin inducible tetO*Mzf1*-*Nkx2.5* CE eGFP ES cell line was established by co-transducing *Nkx2.5* CE eGFP ES cells with tetO*Mzf1*-IRES-puromycin and rtTA lentiviral particles. Transduction was performed with 1000 µl of each lentivirus and additional 500 µl of murine embryonic stem cell medium (without antibiotics) and polybrene (8 µg/ml, Sigma-Aldrich) in 6 cm plates. Fresh virus was added 8 h later. 48 h after transduction doxycyclin (1 µg/ml) (Sigma-Aldrich) was added and antibiotic selection was started with puromycin (1 µg/ml) (PAA). About 10 days post-transduction, colonies were individually expanded still under selection pressure of doxycyclin (dox) and puromycin. The inducibility of *Mzf1* by the tetracyclin responding element (TRE) was confirmed by cultivating one half of each clone for 48 h with dox and the other half without dox. qRT-PCR analysis confirmed the sufficient inducibility of *Mzf1* expression. First differentiation experiments were performed with two of the ES cell lines (clone 44 and 64) to exclude effects caused by different integration sites of the vectors into the genomic sequence. Clone 64 turned out to be more suitable for further studies. For cultivation and differentiation of these cell-lines tetracycline-free FCS was used to exclude cross reactions with the rtTA element.

***Cell Proliferation - MTT-Assays***

The sensitivity of tetO*Mzf1*-*Nkx2.5* CE eGFP ES cells to doxycyclin was evaluated using 3-[4.5-Dimethylthiazol-2-yl]-2.5-diphenyltetrazolium bromide (MTT) [[9](#_ENREF_9)]. Briefly, cells were seeded into 48-well flat-bottomed gelatine coated plates (Greiner Bio One, Frickenhausen, Germany) at a density of 2*10^5^ cells/well (volume 250 µl) and incubated at 37 °C, 5 % CO_2_ for 48 h. Two µg/ml doxycyclin were added for 48 h or five to nine days even before seeding the cells for the MTT-Assay. After incubation for 1 h with 25 µl of 5 mg/ml MTT solution (Sigma) in 225 µl DMEM medium (final concentration 0.5 mg/ml), the medium was aspirated and 250 µl of dimethyl sulfoxide (DMSO, Sigma) were added to each well to dissolve formazan. The well contents were thoroughly mixed and transferred to a 96-well plate (Greiner Bio One) to measure absorbance at 590 nm on an ELISA reader MR 5000. The reference wavelength was set at 690 nm. Three independent experiments were performed.

***Immunocytochemistry***

The maintenance of pluripotency in *Nkx2.5* CE eGFP and dox-inducible tetO*Mzf1-Nkx2.5* CE eGFP ES cells (grown with or without dox) was analyzed with an anti-Sox2 antibody. ES cells were seeded into 4-well chamber slides (BD) on a mitomycin-inactivated MEF feeder layer (density of 4*10^5^ ES cells/well). ES cells were fixed in ice-cold acetone (Sigma) for 10 min at -20°C and then incubated in 0.1 % Triton X-100 (Carl Roth) for 10 min. After blocking with 5 % normal goat serum (Santa Cruz) for 1 h at RT, cells were incubated with the anti-Sox2 antibody (Abcam, ab137385, dilution: 1:250) in 1.5 % normal goat serum at 4°C for 1 h. An appropriate Alexa 488 labeled secondary antibody (goat anti-rabbit IgG, Abcam, ab150077, dilution 1:500) was added for 1 h at RT in the dark. Negative controls were performed with the secondary antibody only. After staining cells were embedded in mounting medium with DAPI to stain the nuclei (Hard Set, Vectashield, H1500).

Furthermore, an efficient *Mzf1* expression induced by the application of dox during *in vitro* differentiation of tetO*Mzf1*-*Nkx2.5* CE eGFP ES cells was validated by immunostaining with an anti-flag antibody. Embryoid bodies (EBs) were seeded into gelatinized 4-well chamber slides. EBs were fixed for 15 min with 4% paraformaldehyde and then permeabilized with 0.25% Triton-X-100 for 10 min. Co-staining with an anti-flag (Sigma, F1804, dilution 1:1000) and an anti-GFP (Abcam, ab6556, dilution 1:2000) antibody was performed for 2 h at 37°C. Appropriate secondary fluorescent antibodies (dilution 1:500) were added for 1 h at RT in the dark and further procedures were accomplished as described above.

All immunostainings were examined by an Axiovert 200M microscope and images were taken with an AxioCam MRm and the AxioVison Rel 4.8 software version 2.0.

***Flow cytometry***

*Nkx2.5* CE eGFP and dox-inducible tetO*Mzf1-Nkx2.5* CE eGFP differentiating ES cells were mechanically detached from cell culture dishes at specific dates and digested with a collagenase II (10000 U/ml, Worthington Biochemical Corporation, Lakewood, NJ)/DNase I (10000 U/µl, Roche) mixture rotating or shaking for 1 h at 37°C. *Nkx2.5* CE eGFP mouse embryos were dissected on E 9.5. Isolated embryos were cut and digested following the above mentioned protocol to obtain a single cell suspension. Adult cardiomyocytes (CMs) were isolated from six week old *αMHC*-Cre/ROSA26^mT/mG^ mice. Animals were euthanized by cervical dislocation after anesthetizing with 2 % isoflurane. The hearts were immediately isolated and placed in cold PBS. Each heart was mechanically dissociated into small pieces followed by enzymatic digestion according to the above mentioned procedure including erythrocyte lysis (Red blood cell lysis solution, Miltenyi Biotec, Bergisch-Gladbach, Germany) and a 70 µm-filtration step to obtain single cell suspension. Dissociated EBs, embryos and heart cells were washed with PBS and resuspended in PBS/0.5 % FCS/2 mM EDTA for flow cytometry. Dead cells were stained with propidium iodide solution (2 µg/ml, Sigma-Aldrich).

Flow cytometry data were acquired using a FACS ARIA^TM^ Illu flow cytometer (BD Biosciences, San Jose, CA) and the BD FACSDiva software version 6.1.2 (BD Biosciences). The results were analyzed with the FlowJo 7.6.5 software (Tree Star, Ashland, OR).

***RNA Isolation and qRT-PCR***

Total RNA was isolated using the peqGold total RNA Kit according to manufacturer’s instructions (Peqlab, Erlangen, Germany) and was reverse transcribed into first strand cDNA with random hexamer primers (Invitrogen) by Omniscript (Qiagen) or MLV reverse transcriptase (Invitrogen). Semiquantitative real time PCR (qRT-PCR) was performed using gene-specific primer sets (ELLA Biotech) with QuantiTect SybrGreen (Qiagen) for 40 cycles. Primer sets are provided in Table 1. ΔCT calculations were performed by a Light Cycler 1.5 (Roche). Each sample was normalized against its *β-Actin* value.

***Protein Isolation and Western blotting***

Whole protein lysates were extracted on day eight of *in vitro* differentiated dox-inducible *Mzf1* over-expressing *Nkx2.5* CE eGFP ES cells. As a positive control protein from a whole mouse heart was extracted. Protein extraction from organs or cells was performed with T-Per Tissue Protein Extraction Buffer (Thermo Fisher Scientific, Bonn, Germany) according to manufacturer’s recommendation. Protein concentration in cell lysates was estimated using the BCA protein assay. Between 40 and 80 µg of whole protein lysates or 5 µl of *in vitro* translated proteins were separated by 10 % SDS-PAGE and transferred to PVDF membranes (BioRad). For the detection of Mzf1, Mesp1 and Tnnt2 membranes were blocked for 1 h in TBS-T (Tris-buffered saline with 0.05 % Tween) containing 5 % nonfat milk (Sigma-Aldrich) followed by rotating overnight-incubation at 4°C with the respective primary antibodies (Mzf1 and Mesp1 *in vitro* translated protein (ANTI-FLAG M2 Antibody, Sigma-Aldrich, 1:5000), Tnnt2 (Abcam, ab10214, 1:250), diluted in blocking reagent). After extensive rinsing incubation with HRP-conjugated secondary antibodies (Millipore) for 90 min at room temperature followed and proteins were visualized by performing luminol enhanced chemiluminescence (WesternBright^TM^ Chemilumineszenz Substrat Sirius, Biozym, Hessisch Oldedorf, Germany).

Loading of equal amounts of proteins was confirmed by reprobing the membranes with an antibody against GAPDH (Millipore). Blots were scanned and analyzed using a gel documentator (ChemiDoc XRS, BioRad) and the Quantity One software (version 4.6.9, BioRad).

***Data Analysis and Statistical Analysis***

All assays were at least performed in triplicates. Data are presented as mean values ± standard error (S.E.M.). Statistical differences were evaluated using the unpaired Student’s t-test or the Mann-Whitney-U test. Comparison of several groups was done by one way ANOVA or the Kruskal-Wallis test on ranks including appropriate post-hoc tests. A value of *p* < 0.05 was considered to be statistically significant. In all figures statistical significance is indicated as follows: * *p* < 0.05 and ** *p* < 0.01.

**REFERENCES**

1. Claycomb WC, Lanson NA, Jr., Stallworth BS, Egeland DB, Delcarpio JB, et al. (1998) HL-1 cells: a cardiac muscle cell line that contracts and retains phenotypic characteristics of the adult cardiomyocyte. Proc Natl Acad Sci U S A 95: 2979-2984.

2. Fatima N, Schooley JF, Jr., Claycomb WC, Flagg TP (2012) Promoter DNA methylation regulates murine SUR1 (Abcc8) and SUR2 (Abcc9) expression in HL-1 cardiomyocytes. PLoS One 7: e41533.

3. Wu SM, Fujiwara Y, Cibulsky SM, Clapham DE, Lien CL, et al. (2006) Developmental origin of a bipotential myocardial and smooth muscle cell precursor in the mammalian heart. Cell 127: 1137-1150.

4. Chen JX, Krane M, Deutsch MA, Wang L, Rav-Acha M, et al. (2012) Inefficient reprogramming of fibroblasts into cardiomyocytes using Gata4, Mef2c, and Tbx5. Circ Res 111: 50-55.

5. Huang X, Wu SM (2010) Isolation and functional characterization of pluripotent stem cell-derived cardiac progenitor cells. Curr Protoc Stem Cell Biol Chapter 1: Unit 1F 10.

6. Dang X, Raffler NA, Ley K (2009) Transcriptional regulation of mouse L-selectin. Biochim Biophys Acta 1789: 146-152.

7. Stadtfeld M, Maherali N, Breault DT, Hochedlinger K (2008) Defining molecular cornerstones during fibroblast to iPS cell reprogramming in mouse. Cell Stem Cell 2: 230-240.

8. Gregoire S, Karra R, Passer D, Deutsch MA, Krane M, et al. (2013) Essential and unexpected role of yin yang 1 to promote mesodermal cardiac differentiation. Circ Res 112: 900-910.

9. Lahm H, Suardet L, Laurent PL, Fischer JR, Ceyhan A, et al. (1992) Growth regulation and co-stimulation of human colorectal cancer cell lines by insulin-like growth factor I, II and transforming growth factor alpha. Br J Cancer 65: 341-346.

10. Lien CL, Wu C, Mercer B, Webb R, Richardson JA, et al. (1999) Control of early cardiac-specific transcription of Nkx2-5 by a GATA-dependent enhancer. Development 126: 75-84.

11. Bondue A, Lapouge G, Paulissen C, Semeraro C, Iacovino M, et al. (2008) Mesp1 acts as a master regulator of multipotent cardiovascular progenitor specification. Cell Stem Cell 3: 69-84.
