# Supplementary material for: Myeloid Zinc Finger 1 (Mzf1) Differentially Modulates Murine Cardiogenesis by Interacting with an Nkx2.5 Cardiac Enhancer
Source: PLoS One. 2014 Dec 1;9(12):e113775. doi: 10.1371/journal.pone.0113775 (PMC4249966; doi:10.1371/journal.pone.0113775)
Supplement: Table S1 — Transcription factor candidates from in silico analysis of the Nx2.5 CE element in alphabetical order. Boldly printed TFs were chosen for further analysis by luciferase reporter assays. (DOCX) [file pone.0113775.s005.docx]

**Supplemental Table S1. Transcription factor candidates from *in silico* analysis of the *Nx2.5* CE element in alphabetical order.** Boldly printed TFs were chosen for further analysis by luciferase reporter assays.

|  | **TF** | **Official name/ also known as** | **TF Search** | **P-Match** | **PROMO3.0** | **JASPAR Database** | **Con Site** | **NCBI summary** |
| --- | --- | --- | --- | --- | --- | --- | --- | --- |
| **1** | **Brachyury** | T, brachyury homolog (mouse)/ TFT | 0 | - | - | 1 | 1 | embryonic nuclear TF; effects transcription of **genes required for mesoderm formation and differentiation**; localized to notochord-derived cells. |
| 2 | Cdx TFs |  |  |  |  |  |  |  |
|  | Cdx-1 | caudal type homeobox 1/ | 0 | - | 54 | - | n.p. | member of the caudal-related homeobox TF gene family; regulates intestine-specific gene expression and enterocyte differentiation; induces expression of the intestinal alkaline phosphatase gene; inhibits beta-catenin/T-cell factor transcriptional activity. |
|  | Cdx-2 | caudal type homeobox/ 2CDX3; CDX-3 | 0 | - | - | - | n.p. | member of the caudal-related homeobox TF gene family; major regulator of intestine-specific genes involved in cell growth an differentiation; also plays a role in early embryonic development of the intestinal tract; aberrant expression is associated with intestinal inflammation and tumorigenesis. |
|  | CdxA |  | 119 | 110 | - | - | n.p. |  |
| 3 | Ets TFs |  |  |  |  |  |  |  |
|  | C-Ets (Ets1) | v-ets avian erythroblastosis virus E26 oncogene homolog 1/ ETS-1; EWSR2 | 7 | 12 | 4/23 | 70 | 70 | erythroblast transformation-specific (ETS) family of TFs; required for platelet adhesion to the subendothelium, inducing vascular cell remodeling; also regulates hematopoiesis, and the differentiation and maturation of megakaryocytic cells. |
|  | C-Ets (Ets2) | v-ets avian erythroblastosis virus E26 oncogene homolog 2/ ETS2IT1 | 7 | 0 | 6 | - | 70 | regulates genes involved in development and apoptosis; protooncogene and shown to be involved in regulation of telomerase |
| 4 | CP2 | TF CP2, tfcp2 | 2 | 1 | 27 | - | n.p. | No summary |
| 5 | E2F |  | 3 | 1 | 7/3 | 4 | 4 |  |
|  | E2F1 | E2F TF 1; RBP3; RBAP1; RBBP3 | - | 0 | 34/16 | 4 | - | member of the E2F family of TFs; E2F family plays a crucial role in the control of cell cycle and action of tumor suppressor proteins and is also a target of the transforming proteins of small DNA tumor viruses; binds preferentially to retinoblastoma protein pRB in a cell-cycle dependent manner; can mediate both cell proliferation and p53-dependent/independent apoptosis. |
| **6** | **Elk1** | ELK1, member of ETS oncogene family/ | 2 | 2 | 109 | 14 | 14 | member of the Ets family of TFs and of the ternary complex factor (TCF) subfamily; forms a ternary complex by binding to the the serum response factor and the serum response element in the promoter of the c-fos proto-oncogene; a nuclear target for the ras-raf-MAPK signaling cascade; |
| 7 | En1 | engrailed homeobox 1/ | n.p. | 91 | 17 | 23 | 23 | role in controlling development; in Drosophila: plays an important role during development in segmentation; required for the formation of posterior compartments; different mutations in the mouse homologs (En1, En2) produced different developmental defects (frequently lethal); human homologs (EN1, EN2) have been implicated in the control of pattern formation during development of the central nervous system. |
| 8 | Evi1/Runx1 | runt-related transcription factor 1/ AML1; CBFA2; AMLCR1; PEBP2aB; AML1-EVI-1, | 4 | 123 | 2 | 2 | 2 | heterodimeric TF that binds to the core element of many enhancers and promoters; thought to be involved in the development of normal hematopoiesis; chromosomal translocations involving this gene are well-documented and have been associated with several types of leukemia. |
| 9 | FOXD3 | forkhead box D3/ AIS1; **HFH2**; VAMAS2; Genesis | 4 | 15 | - | 17 | 17 | belongs to the forkhead family of TFs; mutations cause autoimmune susceptibility 1. |
| **10** | **Gata TFs** |  |  |  |  |  |  |  |
|  | Gata1 | GATA binding protein 1 (globin TF 1)/ GF1; NFE1; XLTT; ERYF1; **NF-E1**; XLANP; XLTDA | 30 | - | 32 | 12 | 54 | Belongs to the GATA family of TFs; important role in erythroid development by regulating the switch of fetal hemoglobin to adult hemoglobin; mutations in this gene have been associated with X-linked dyserythropoietic anemia and thrombocytopenia. |
|  | Gata2 | GATA binding protein 2/ DCML; NFE1B; MONOMAC | 11 | 0 | 4/7 | 4 | 68 | Belongs to the GATA family of zinc-finger TFs, essential role in regulating transcription of genes involved in the development and proliferation of hematopoietic and endocrine cell lineages. |
|  | Gata3 | GATA binding protein 3/ HDR; HDRS | 6 | 0 | 6 | 10 | 48 | Belongs to the GATA family of TFs; important regulator of T-cell development; plays an important role in endothelial cell biology; defects in this gene cause hypoparathyroidism with sensorineural deafness and renal dysplasia. |
|  | **Gata4** | GATA binding protein 4/ ASD2; VSD1 | n.p. | 0 | - | 15 | n.p. | member of the GATA family of zinc-finger TFs; thought to regulate genes involved in embryogenesis, in **myocardial differentiation** **and function;** mutations in this gene have been associated with **cardiac septal defects**. |
| **11** | **Hand1** | heart and neural crest derivatives expressed 1/ Hxt; eHand; **Thing1**; bHLHa27 | n.p. | 0 | - | 11 | 11 | belongs to the basic helix-loop-helix family of TFs; one of two closely related family members, the HAND proteins, which are asymmetrically expressed in the developing ventricular chambers and play an **essential role in cardiac morphogenesis**; in a complementary fashion, they function in the **formation of the right ventricle and aortic arch arteries**, implicating them as mediators of congenital heart disease; may be also required for early trophoblast differentiation. |
| 12 | HOXA3 | homeobox A3/ HOX1; HOX1E | n.p. | 300 | 84 | 2 | n.p. | class of TFs called homeobox genes; found in clusters named A, B, C, and D on four separate chromosomes; spatially and temporally regulated during embryonic development; may regulate gene expression, morphogenesis, and differentiation. |
| **13** | **Klf4** | Kruppel-like factor 4 (gut)/ EZF; GKLF | n.p. | - | - | 64 | n.p. | No summary |
| 14 | Lyf-1 (IKZF1) | IKAROS family zinc finger 1 (Ikaros)/ **IK1**; hIk-1; IKAROS; PRO0758; ZNFN1A1 | 2 | 10 | 11/3 | - | n.p. | belongs to the family of zinc-finger DNA binding proteins associated with chromatin remodeling; restricted to the fetal and adult hemo-lymphopoietic system; functions as a regulator of lymphocyte differentiation; overexpression of some dominant-negative isoforms have been associated with Bcell malignancies, such as acute lymphoblastic leukemia. |
| **15** | **Mesp1** | mesoderm posterior 1 homolog (mouse)/ bHLHc5 | n.p. | n.p. | n.p. | n.p. | n.p. | No summary |
| **16** | **Msx1** | msh homeobox 1/ HOX7; HYD1; ECTD3; STHAG1 | 0 | 218 | 89 | 2 | n.p. | member of the muscle segment homeobox gene family; transcriptional repressor during embryogenesis through interactions with components of the core transcription complex and other homeoproteins; roles in limb-pattern formation, craniofacial development, particularly odontogenesis, and tumor growth inhibition; mutations in this gene have been associated with nonsyndromic cleft lip with or without cleft palate 5, Witkop syndrome, Wolf-Hirschom syndrome, and autosomoal dominant hypodontia |
| 17 | MyoD | myogenic differentiation 1/ PUM; MYF3; MYOD1; bHLHc1 | 3 | 0 | 20 | 3 | 3 | basic helix-loop-helix family of TFs and the myogenic factors subfamily; regulates muscle cell differentiation by inducing cell cycle arrest, a prerequisite for myogenic initiation; is also involved in muscle regeneration. |
| **18** | **Myogenin** | myogenin (myogenic factor 4)/ **MYF4**; myf-4; bHLHc3 | 0 | 7 | 34 | 3 | 3 | member of the helix-loop-helix (HLH) proteins; essential for the development of functional skeletal muscle; muscle-specific TF that can induce **myogenesis** in a variety of cell types in tissue culture |
| **19** | **Mzf1** | myeloid zinc finger 1/ MZF1B, ZFP98, ZNF42, ZSCAN6; | 22 | 39 | 19 | 92 | 92 | No summary |
| 20 | NF-E2 | nuclear factor, erythroid 2/ p45; NF-E2 | 3 | 1 |  | - | n.p. | No summary |
| 21 | Oct1 (POU2F1) | POU class 2 homeobox 1/ OTF1; oct-1B | 5 | 49 | 17/3 | - | n.p. | member of the POU TF family; |
| 22 | p300 | E1A binding protein p300/ EP300; KAT3B; RSTS2 | 3 | 32 | 109 | - | n.p. | transcriptional co-activator protein; functions as histone acetyltransferase that regulates transcription via chromatin remodeling and is important in the processes of cell proliferation and differentiation; mediates cAMP-gene regulation by binding specifically to phosphorylated CREB protein; has also been identified as a co-activator of HIF1A (hypoxia-inducible factor 1 alpha), and thus plays a role in the stimulation of hypoxia-induced genes such as VEGF; defects are a cause of Rubinstein-Taybi syndrome and may also play a role in epithelial cancer. |
| 23 | Pax TFs |  |  |  |  |  |  |  |
|  | Pax2 | paired box 2/ PAPRS | 0 | 300 | 18 | 49 | 49 | believed to be a target of transcriptional suppression by the tumor suppressor gene WT1; mutations within PAX2 have been shown to result in optic nerve colobomas and renal hypoplasia. |
|  | Pax4 | paired box 4/ KPD; MODY9 | n.p. | - | 25 | 1 | 1 | member of the paired box (PAX) family of TFs; involved in pancreatic islet development; mouse studies have demonstrated a role in differentiation of insulin-producing beta cells. |
|  | Pax5 | paired box 5/ BSAP | 0 | - | 24/15 | 6 | n.p. | member of the paired box (PAX) family of TFs; encodes the B-cell lineage specific activator protein that is expressed at early, but not late stages of B-cell differentiation; expression has also been detected in developing CNS and testis; may therefore also play a role in neural development and spermatogenesis. |
|  | Pax6 | paired box 6/ AN; AN2; MGDA; WAGR; D11S812E | 0 | 2 | 4/72 | 0 | 0 | expressed in the developing nervous system, and in developing eyes; mutations in this gene are known to cause ocular disorders such as aniridia and Peter's anomaly. |
|  | Pax8 | paired box 8/ | 0 | 0 | 20 | n.p. | n.p. | member of the paired box (PAX) family of TFs; involved in thyroid follicular cell development and expression of thyroid-specific genes; mutations have been associated with thyroid dysgenesis, thyroid follicular carcinomas and atypical follicular thyroid adenomas. |
|  | Pax9a | paired box 9/ STHAG3 | n.p. | - | 29 | n.p. | n.p. | member of the paired box (PAX) family of TFs; specific function of the paired box 9 gene is unknown but it may be involved in development of stratified squamous epithelia as well as various organs and skeletal elements. |
| 24 | Pbx-1 | pre-B-cell leukemia homeobox 1/ | 4 | 35 | 1 | 2 | 2 | PBX homeobox family of TFs, may be involved in the regulation of osteogenesis; required for skeletal patterning and programming; chromosomal translocation, t(1;19) involving this gene and TCF3/E2A gene, is associated with pre-B-cell acute lymphoblastic leukemia. |
| **25** | **Sox-TFs** |  |  |  |  |  |  |  |
|  | Sox13 | SRY (sex determining region Y)-box 13/ ICA12; Sox-13 | n.p. | - | 6 | n.p. | n.p. | member of the SOX (SRY-related HMG-box) family of TFs; may act as a transcriptional regulator after forming a protein complex with other proteins; type-1 diabetes autoantigen, also known as islet cell antibody 12. |
|  | **Sox17** | SRY (sex determining region Y)-box 17/ VUR3 | n.p. | - | - | 14 | 14 | member of the SOX (SRY-related HMG-box) family of TFs; may act as a transcriptional regulator after forming a protein complex with other proteins. |
|  | Sox2 | SRY (sex determining region Y)-box 2/ ANOP3; MCOPS3 | 0 | 0 | 17 | 5 | n.p. | intronless gene; member of the SRY-related HMG-box (SOX) family of TFs; required for stem-cell maintenance in the central nervous system; also regulates gene expression in the stomach; mutations have been associated with optic nerve hypoplasia and with syndromic microphthalmia, a severe form of structural eye malformation. |
|  | Sox5 | SRY (sex determining region Y)-box 5/ L-SOX5 | 1 | - | - | 17 | 18 | member of the SOX (SRY-related HMG-box) family of TFs; may act as a transcriptional regulator after forming a protein complex with other proteins; may play a role in chondrogenesis. |
|  | Sox9 | SRY (sex determining region Y)-box 9/ CMD1; SRA1; CMPD1 | 0 | 2 | - | 4 | 4 | HMG-box class DNA-binding protein; acts during chondrocyte differentiation and, with steroidogenic factor 1, regulates transcription of the anti-Muellerian hormone (AMH) gene; deficiencies lead to the skeletal malformation syndrome campomelic dysplasia, frequently with sex reversal. |
| 26 | Sp1 | specificity protein 1 TF/ | 6 | 34 | 11/2 | 29 | 26 | zinc finger TF; involved in many cellular processes, including cell differentiation, cell growth, apoptosis, immune responses, response to DNA damage, and chromatin remodeling; can be an activator or a repressor. |
| 27 | SRY | sex determining region Y/ SRXX1, SRXY1, TDF, TDY | 11 | 35 | 17 | 23 | 23 | member of the high mobility group (HMG)-box family; the testis-determining factor (TDF) initiates male sex determination; mutations give rise to XY females with gonadal dysgenesis (Swyer syndrome); translocation of part of the Y chromosome containing this gene to the X chromosome causes XX male syndrome. |
| 28 | v-Myb | v-myb avian myeloblastosis viral oncogene homolog/ efg; **c-myb**; c-myb_CDS; MYB | 1 | 45 | 10 | 9 | 10 | member of the MYB family of TF genes; involved in transcriptional activation and repression; plays an essential role in the regulation of hematopoiesis and may play a role in tumorigenesis. |
| 29 | YY1 | YY1 TF/ DELTA; NF-E1; UCRBP; INO80S; YIN-YANG-1 | 0 | 47 | 14/26 | 16 | 16 | ubiquitously distributed TF belonging to the GLI-Kruppel class of zinc finger proteins; repressing and activating diverse promoters; may implicate histone modification in the function of YY1 |

### Abbreviations: TF transcription factor; NCBI National Center for Biotechnology Information (www.ncbi.nlm.nih.gov/); n.p. no matrix profile for the specific TF in the specific transcription factor database;

Promo3.0: there are several matrices for one TF (🡪 therefore different numbers for one TF are possible)
