# Supplementary material for: Myeloid Zinc Finger 1 (Mzf1) Differentially Modulates Murine Cardiogenesis by Interacting with an Nkx2.5 Cardiac Enhancer
Source: PLoS One. 2014 Dec 1;9(12):e113775. doi: 10.1371/journal.pone.0113775 (PMC4249966; doi:10.1371/journal.pone.0113775)
Supplement: Table S2 — Sequences of primer-sets for gene expression analysis by qRT-PCR. (DOCX) [file pone.0113775.s006.docx]

**Supplemental Table S2.** **Sequences of primer-sets for gene expression analysis by qRT-PCR**

| gene | forward (5’ 🡪 3’) | reverse (5’ 🡪 3’) | fragment size |
| --- | --- | --- | --- |
| β-Actin | ccaaccgtgaaaagatgacc | accagaggcatacagggaca | 97 nt |
| Mzf1 | agggggcatcttttcacc | gaggctagccatgtccagat | 112 nt |
| Nkx2.5 | gagcctacggtgaccctga | gtggtctctcggcgccat | 116 nt |
| Tbx5 | ggatgtctcggatgcaaagt | ggttggaggtgactttgtgc | 104 nt |
| Mef2c | atgggcggagatctgaca | ttcttgttcaggttaccaggtg | 107 nt |
| Isl1 | ccacgatgtggtggagaga | ctagccgagatgggttcg | 102 nt |
| Gata4 | ggaagacaccccaatctcg | catggccccacaattgac | 71 nt |
| αActin | ggtcatcaccattggcaac | atgccagcagattccatacc | 83 nt |
| Tnnt2 | ttcgacctgcaggaaaagtt | cttcccacgagttttggaga | 99 nt |
| αMHC | cctatgcttctgctgataccg | tcatcagcttgttcagattttcc | 113 nt |
| Runx1 | ctccgtgctacccactcact | atgacggtgaccagagtgc | 101 nt |
| Sox17 | caacgcagagctaagcaaga | ttgtagttggggtggtcctg | 126 nt |
| Nestin | tgcaggccactgaaaagtt | ttccaggatctgagcgatct | 89 nt |
| Mesp1 | ggcaccaaccggccagaa | aggttgtcctcgctgagtc | 101 nt |
| Flk1 | cagtggtactggcagctagaag | acaagcatacgggcttgttt | 63 nt |
| Tal1 | gctcgccctcactaggcagt | ctcttcacccggttgttgtt | 77 nt |
| Gata1 | gcatcaacaagcccaggt | aaactggggcaagggttc | 88 nt |

Abbreviations: nt = nucleotides
